# Reducing Smoking Requirements for Lung Screening to Address Health Disparities in a Community Cohort

**DOI:** 10.1001/jamanetworkopen.2025.17149

**Published:** 2025-06-24

**Authors:** Matthew P. Smeltzer, Wei Liao, Jordan Goss, Talat Qureshi, Sara Johnson, Amanda Harris, Kourtney Dortch, Carrie Fehnel, Sora Ely, Meredith Ray, Raymond U. Osarogiagbon

**Affiliations:** 1 Division of Epidemiology, Biostatistics, and Environmental Health, School of Public Health, University of Memphis, Memphis, Tennessee; 2 Thoracic Oncology Research Group, Multidisciplinary Thoracic Oncology Program, Baptist Cancer Center, Memphis, Tennessee; 3 George Washington University Hospital, Thoracic Surgery, Washington, DC

## Abstract

**Importance:**

Many individuals with lung cancer are not eligible for lung cancer screening (LCS). Race-based and sex-based differences in smoking patterns reduce the effectiveness of LCS criteria.

**Objective:**

To assess the value of expanding LCS criteria beyond US Preventive Services Task Force (USPSTF) 2021 guidelines by relaxing cigarette smoking exposure criteria.

**Design, Setting, and Participants:**

This cohort study included individuals enrolled in an incidental pulmonary nodule (IPN) program with non–screening-detected, potentially malignant pulmonary nodules, or enrolled in a LCS program from 2015 to 2023. Participants were treated in a community-based health care system serving more than 125 counties in Mississippi, Tennessee, Arkansas, Kentucky, Missouri, and Alabama. Data were examined from October 1, 2024, to May 29, 2025.

**Exposures:**

LCS eligibility criteria included USPSTF 2021, Potter criteria (20-year smoking history), and American Cancer Society criteria ([ACS] 20 pack-years, no quit duration), and expansions to 10 years or 10 pack-years.

**Main Outcomes and Measures:**

The main outcomes were proportions of individuals eligible for LCS and diagnosis of lung cancer. Characteristics between groups were compared using the χ^2^ test for categorical variables and the Wilcoxon-Mann-Whitney test for continuous variables.

**Results:**

In this study, 43 521 individuals were evaluated, including 13 770 (32%) from LCS and 29 751 (68%) from IPN programs. Of the 29751 individuals in IPN programs, 3840 (13%) were eligible for LCS by USPSTF 2021 criteria, 4905 (16%) by IPN-Potter criteria, 5263 (18%) by IPN-ACS criteria, and 6307 (21%) by IPN-Potter-ACS criteria. There were 1103 additional individuals eligible by IPN-Potter criteria (606 female [55%] and 382 Black [35%]), 1423 by IPN-ACS criteria (628 female [44%]; 237 Black [17%]), and 2467 by IPN-Potter-ACS criteria (1200 female [49%]; 615 Black [25%]). There were significant differences in the number of additional eligible individuals who were female (55% vs 48%; *P* < .001) and Black (35% vs 22%; *P* < .001) between the IPN-Potter and USPSTF 2021 criteria. Lung cancer was diagnosed in 504 of 13 770 of LCS enrollees (4%) and 1714 of 29 751 of IPN enrollees (6%), including 872 of 4905 (18%) eligible by IPN-Potter criteria, 955 of 5263 (18%) by IPN-ACS criteria, and 1051 of 6307 (17%) by IPN-Potter-ACS criteria. The additional eligible individuals with lung cancer in IPN programs who were female included 70 of 128 (55%) by IPN-Potter criteria, 89 of 208 (43%) by IPN-ACS criteria, and 143 of 304 (47%) by IPN-Potter-ACS criteria, with statistically significant differences between the IPN-Potter criteria and USPSTF 2021 criteria (55% vs 48%; *P* < .001). The additional eligible individuals who were Black included 50 of 128 (39%) by IPN-Potter criteria, 32 of 208 (15%) by IPN-ACS criteria, and 73 of 304 (24%) by IPN-Potter-ACS criteria, with statistically significant differences between IPN-Potter criteria and IPN-USPSTF 2021 criteria (39% vs 22%; *P* < .001). When LCS criteria were expanded to individuals older than 50 years who had smoked for 10 years or 10 pack-years, 7993 of 29 751 individuals (27%) from IPN programs would have been eligible. Among these, 1251 of 7993 (16%) were diagnosed with lung cancer.

**Conclusions and Relevance:**

In this cohort study, changing smoking criteria from 20 pack-years to a 20-year smoking history was associated with improved access to LCS while maintaining diagnostic efficiency. Expansion to individuals with a 10-year or 10 pack-years smoking history should be explored.

## Key Points

**Question:**

Will relaxing the cigarette smoking criteria for lung cancer screening reduce race- and sex-based disparities and identify more lung cancers, without a considerable loss of efficiency?

**Findings:**

In this cohort study of 43 521 individuals, changing lung screening criteria from 20 pack-years to 20 years of smoking was associated with an increase in the number of individuals who were eligible and reduced race- and sex-based disparities in eligibility, while maintaining diagnostic efficiency. Further expansion to individuals with 10 years of smoking or 10 pack-years continued to identify lung cancer in high proportions in this cohort.

**Meaning:**

These findings suggest that the expansion of lung cancer screening eligibility to individuals with less smoking exposure may be justified.

## Teaser

This cohort study estimates the potential benefit of expanding lung screening criteria by relaxing cigarette smoking exposure criteria to reduced race- and sex-based disparities and identify more lung cancers while maintaining diagnostic efficiency.

## Introduction

Lung cancer screening (LCS) reduces mortality, but adoption has been slow for many reasons, including the complexity of eligibility criteria, especially with regard to the smoking history.^1,2,3,4^ In the US, LCS eligibility entirely depends on age and cigarette smoking history.^1^ US Preventive Services Task Force (USPSTF) 2021 eligibility criteria exclude almost half of persons diagnosed with lung cancer.^5,6^ However, the current screening eligibility criteria are derived from clinical trials designed to test the hypothesis that LCS by low-dose computed tomography reduces mortality.^3,4^ The trials were not designed to establish the optimal at-risk population for program implementation. With the hypothesis empirically proven, there is now a public health need to optimize eligibility criteria to fit the broader population at risk, thereby expanding the reach of early lung cancer detection.^7^

Race-based and sex-based differences in smoking patterns, including mean smoking duration, the number of cigarettes smoked per day, as well as susceptibility to lung cancer at lower levels of cigarette use, influence lung cancer risk and screening eligibility.^8,9^ Evidence from multiple studies suggests that the USPSTF 2013 guidelines inadvertently induced race-based and sex-based disparities in LCS eligibility and access.^7,10,11^ For example, evaluation of a large southern US cohort revealed that among individuals who had a history of smoking and had lung cancer, 56% of White individuals were eligible by USPSTF 2013 criteria compared with 32% of Black individuals.^10^ The USPSTF 2021 criteria were designed to address this problem by reducing the age of eligibility from 55 years to 50 years, and the smoking intensity requirement from 30 pack-years to 20 pack-years.^5^ Nevertheless, race-based disparities persist.^8,10,12^ Therefore, LCS eligibility criteria must continue to evolve toward greater equity, with the aim of identifying more lung cancers early and reducing disparities, while maintaining the efficiency and safety of screening. Recently proposed additional modifications of the cigarette smoking requirements include removing the quit duration constraint, considering years smoked rather than pack-years, or using risk calculators, such as the PLCO_m2012_, which include additional risk factors.^13,14,15^

We previously demonstrated the improved performance of USPSTF 2021 over USPSTF 2013 screening criteria in a Mississippi Delta population involving LCS and incidental pulmonary nodule (IPN) cohorts.^12,16^ We now assess the potential value of expanding LCS criteria beyond USPSTF 2021 using updated data from these cohorts. We hypothesized that relaxing the cigarette smoking exposure criteria would identify more early-stage lung cancers, without a considerable loss of efficiency.

## Methods

This cohort study was approved by the institutional review board at Baptist Memorial Healthcare Corporation and followed the Strengthening the Reporting of Observational Studies in Epidemiology (STROBE) reporting guideline. The requirement of informed consent was waived because the study is quality improvement and low risk. In 2015, we constructed 2 parallel prospective observational datasets including all patients enrolled into the institution’s low-dose computed tomography LCS and IPN programs. Patients in the combined programs constitute the ongoing Detecting Early Lung Cancer (DELUGE) in the Mississippi Delta cohort.^16,17^ The institution’s service area population includes more than 125 counties in Mississippi, western Tennessee, eastern Arkansas, southwestern Kentucky, southeastern Missouri, and northwestern Alabama.

Eligibility for the screening program is based on existing Medicare and USPSTF criteria.^5,18,19^ The IPN program includes individuals with nonscreening, incidentally detected, potentially malignant pulmonary nodules identified through an automated process from radiology reports. Radiologists included a standardized statement on reports when they detected a pulmonary lesion that, based on their clinical judgement, required further evaluation. The screening program uses Lung Imaging and Reporting Data System (Lung-RADS) and the IPN program uses Fleischner Society guidelines for risk stratification, and recommendations for lesion management. DELUGE includes demographics, clinical characteristics, procedures, complications, and health outcomes. Data reside in a secure Research Electronic Data Capture (REDCap) database. Trained full-time data managers individually abstract, manage, audit, and update data for every individual in the DELUGE Cohort, using our standard operating procedures.^16^

### Screening Eligibility Groups and Group Comparisons

We compared individuals in the DELUGE LCS and IPN cohorts who would have been eligible for LCS by various criteria, or ineligible (not eligible by any criteria including those with no documented smoking history).^5,13,20^ The screening criteria groups included LCS-Eligible, LCS-Eligible-RADS 3-4, IPN-Screening Ineligible, IPN-USPSTF 2021, IPN-Potter, IPN-ACS, and IPN-Potter-ACS (Table 1). We also evaluated additional persons eligible for screening, who were not eligible by USPSTF 2021 but would have been rendered eligible by each of the new criteria (labeled as IPN-Potter Extra, IPN-ACS Extra, and IPN-Potter-ACS Extra).

**Table 1.** Screening Criteria Groups Evaluated

Criteria group	Eligibility description
LCS-Eligible	Patients who received lung cancer screening and were eligible by USPSTF 2021 criteria
LCS-Eligible-RADS 3-4	Patients who received lung cancer screening and were eligible by USPSTF 2021 criteria and had a Lung-RADS score of 3 or 4.
IPN-Screening ineligible	Patients in the DELUGE IPN who were ineligible for screening by all criteria evaluated.
IPN-USPSTF 2021	Patients in the DELUGE IPN who were eligible for screening by USPSTF 2021 (age 50-80, 20 pack-year history, quit duration less than 15 years)
IPN-Potter	Patients in the DELUGE IPN eligible by with Potter criteria (age 50 to 80 years, 20 years smoking duration, quit duration less than 15 years)^13^
IPN-ACS	Patients in the DELUGE IPN eligible by new ACS criteria (age 50 to 80 years, 20 pack year history, no quit duration requirement)
IPN-Potter-ACS	Patients in the DELUGE IPN eligible by ACS or Potter (age 50 to 80 years, 20 pack-year history or 20 year smoking duration, no quit duration requirement)

Abbreviations: ACS, American Cancer Society; DELUGE, Detecting Early Lung Cancer; IPN, incidental pulmonary nodule; LCS, lung cancer screening; RADS, reporting and data system; USPSTF, United States Preventive Services Task Force.

We compared individual eligibility groups by sex, self-identified race, patient-level rurality (county-level Rural-Urban Continuum Area Codes), patient-level Area Deprivation Index (ADI), smoking exposure, comorbidities, lung cancers diagnosed, and number of eligible individuals for every lung cancer identified. In those diagnosed with lung cancer, we compared histologic type, tumor size, and clinical staging.

### Statistical Analysis

We report summary statistics including mean (SD) and median (IQR). Follow-up time was calculated from cohort enrollment to lung cancer diagnosis or last follow-up. Secondary analysis limited the cohort to individuals with at least 24 months of follow-up. We compare characteristics between groups using the χ^2^ test for categorical variables and the Wilcoxon-Mann-Whitney test for continuous variables. Analyses were conducted with R version 4.4.1 (R Project for Statistical Computing) using an α = .05 to determine statistical significance without adjusting for multiple comparisons. Tests were 2-sided. Data were analyzed from October 1, 2024, to May 29, 2025.

## Results

We evaluated 43 521 individuals in the DELUGE cohort from 2015 to 2023, which included 13 770 from LCS (32%) and 29 751 from IPN (68%). Among the 13 770 individuals in the LCS cohort, 6784 were females (49%), 2841 were Black individuals (21%), 10 595 were White individuals (77%); the median (IQR) age was 65 (59-69) years and median (IQR) follow-up time was 2.2 (1.0 to 4.0) years (Table 2). In the LCS cohort, 5252 individuals were rural residents (38%), and lung cancer was diagnosed with 504 individuals (4%). Among the 29 751 individuals in the IPN cohort, 16 599 were females (56%), 8626 were Black individuals (29%), 19 992 were White individuals (67%); the median (IQR) age was 64 (53-74) years and the median follow-up time was 2.5 (1.0 to 5.0) years (Table 2). In the IPN cohort, 7181 were rural residents (24%) and lung cancer was diagnosed in 1714 individuals (6%).

**Table 2.** Demographics by Cancer Status for Incidental Pulmonary Nodule (IPN) and Lung Cancer Screening (LCS) Cohorts

Demographic characteristic	Patient, No. (%)					
	LCS			IPN		
	Total (n = 13 770)	Cancer (n = 504)	Noncancer (n = 13 266)	Total (n = 29 751)	Cancer (n = 1714)	Noncancer (n = 28 037)
Lung cancer	504 (4)	504 (100)	0	1714 (6)	1714 (100)	0
Screen age						
Mean (SD)	65 (7)	68 (6)	64 (7)	63 (15)	70 (10)	62 (16)
Median (IQR) [range]	65 (59-69) [26-94]	68 (64-72) [50-87]	65 (59-69) [26-94]	64 (53-74) [1-107]	70 (63-76) [33-100]	64 (52-74) [1-107]
Sex						
Male	6985 (51)	244 (48)	6741 (51)	13 150 (44)	858 (50)	12 292 (44)
Female	6784 (49)	260 (52)	6524 (49)	16 599 (56)	855 (50)	15 744 (56)
Missing	1 (0.01)	0	1 (0.01)	2 (0.01)	1 (0.06)	1 (0.01)
Race						
Black	2841 (21)	88 (17)	2753 (21)	8626 (29)	433 (25)	8193 (29)
White	10 595 (77)	410 (81)	10 185 (77)	19 992 (67)	1252 (73)	18 740 (67)
Other or unknown^a^	334 (2)	6 (1)	328 (2)	1133 (4)	29 (2)	1104 (4)
Ethnicity						
Hispanic or Latino	67 (0.5)	1 (0.2)	66 (0.5)	526 (1.8)	11 (0.6)	515 (1.8)
Not Hispanic or Latino	13 371 (97)	492 (98)	12 879 (97)	28 765 (97)	1689 (99)	27 076 (97)
Other or unknown	332 (2)	22 (2)	321 (2)	460 (2)	14 (1)	446 (2)
Insurance						
Medicare	7808 (57)	357 (71)	7451 (56)	14 745 (50)	1194 (70)	13 551 (48)
Medicaid	507 (4)	16 (3)	491 (4)	946 (3)	48 (3)	898 (3)
Commercial	5245 (38)	128 (25)	5117 (39)	11 860 (40)	398 (23)	11 462 (41)
Self-insured or unknown	210 (2)	3 (1)	207 (2)	2200 (7)	74 (4)	2126 (8)
RUCA code						
Missing	2 (0.01)	0	2 (0.02)	25 (0.08)	0	25 (0.09)
Metro area	8516 (62)	334 (66)	8182 (62)	22 545 (76)	1258 (73)	21 287 (76)
Rural area	5252 (38)	170 (34)	5082 (38)	7181 (24)	456 (27)	6725 (24)
ADI quintile, %						
Missing	2 (0.01)	0	2 (0.02)	26 (0.09)	0	26 (0.09)
Q1, 0-20	212 (2)	4 (1)	208 (2)	587 (2)	27 (2)	560 (2)
Q2, 21-40	823 (6)	24 (5)	799 (6)	2671 (9)	138 (8)	2533 (9)
Q3, 41-60	2900 (21)	116 (23)	2784 (21)	6797 (23)	389 (23)	6408 (23)
Q4, 61-80	4121 (30)	148 (29)	3973 (30)	8929 (30)	532 (31)	8397 (30)
Q5, 81-100	5712 (41)	212 (42)	5500 (41)	10 741 (36)	628 (37)	10 113 (36)

Abbreviations: ADI, Area Deprivation Index; RUCA, Rural-Urban Continuum Area; Q, quintile.

^a^ Individuals with race Asian, American Indian or Alaska Native, Native Hawaiian or Pacific Islander, or unknown.

### Screening Eligibility

**Figure 1.**
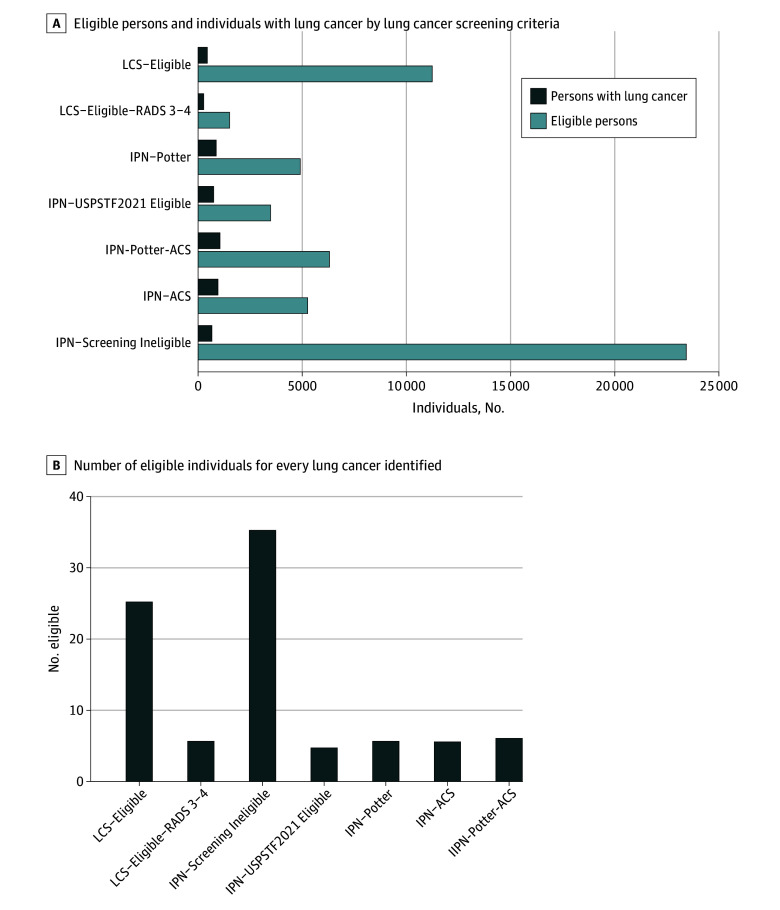
Number of Eligible Persons and Individuals With Lung Cancer by Lung Cancer Screening (LCS) Eligibility and Criteria ACS indicates American Cancer Society; IPN, incidental pulmonary nodule; RADS, reporting and data systems; USPSTF, United States Preventive Services Task Force.

We compared 7 groups from the DELUGE dataset, based on the screening eligibility criteria applied, 2 from LCS (included for comparison) and 5 from the IPN program (Table 1 and eFigure 1 in Supplement 1). LCS-eligible includes all 11 244 individuals who received LCS and were eligible for screening by USPSTF 2021. The LCS-USPSTF21-RADS3-4 is a subset of LCS-eligible consisting of 1510 individuals who received LCS (13%), were eligible for screening by USPSTF 2021, and had a Lung-RADS score of 3 or 4, indicating they had a potentially malignant lung lesion (Table 1 and Figure 1).

The IPN groups had non–screening-detected pulmonary nodules and did not participate in LCS. From the IPN cohort, we found IPN-USPSTF 2021 was the most restrictive, with 3840 of 29 751 eligible individuals (13%). IPN groups with relaxed eligibility criteria by smoking status had increasing numbers of individuals eligible: 4905 with the IPN-Potter criteria (17%), 5263 with the IPN-ACS criteria (18%), and 6307 with the IPN-Potter-ACS criteria (21%) (Figure 1). However, 23 444 of 29 751 individuals (79%) in the IPN cohort were ineligible for LCS by all criteria (IPN-screening ineligible). The median (IQR) age was between 65 (59 to 69) years and 67 (61 to 73) years for all groups (eTable 1 in Supplement 1). The percentage female was 47% to 50% (2475 of 5263 for IPN-ACS criteria; 2442 of 4905 for IPN-Potter criteria; 3047 of 6307 for IPN-Potter-ACS criteria; 1847 of 3840 for IPN-USPSTF 2021) in varying IPN screening eligible groups but was higher in IPN-screening ineligible (13 552 of 23 444 [58%]; *P* < .001). Similarly, the percentage of Black individuals was 19% to 24% (1014 of 5263 for IPN-ACS criteria; 1154 of 4905 for IPN-Potter criteria; 1392 of 6307 for IPN-Potter-ACS criteria; 777 of 3840 for IPN-USPSTF 2021) across IPN eligible groups but was higher in IPN-screening ineligible (7234 of 23 444 [31%]; *P* < .001). Additionally, 27% to 29% (1466 of 5263 for IPN-ACS criteria; 1395 of 4905 for IPN-Potter criteria; 1730 of 6307 for IPN-Potter-ACS criteria; 1123 of 3840 for IPN-USPSTF 2021) had rural residence across IPN eligible groups but this was lower in the IPN-Screening Ineligible group (5451 of 23 444 [23%]; *P* < .001). ADI is presented in quartiles by patient ZIP code in eFigure 2 in Supplement 1. The total proportions of the 1714 persons diagnosed with lung cancer in IPN who would have been screening eligible included 747 individuals (44%) with the USPSTF 2021 criteria, 872 (51%) with the IPN-Potter criteria, 955 (56%) with the IPN-ACS criteria, and 1051 (61%) with the IPN-Potter-ACS criteria.

### Additional Persons Eligible

**Figure 2.**
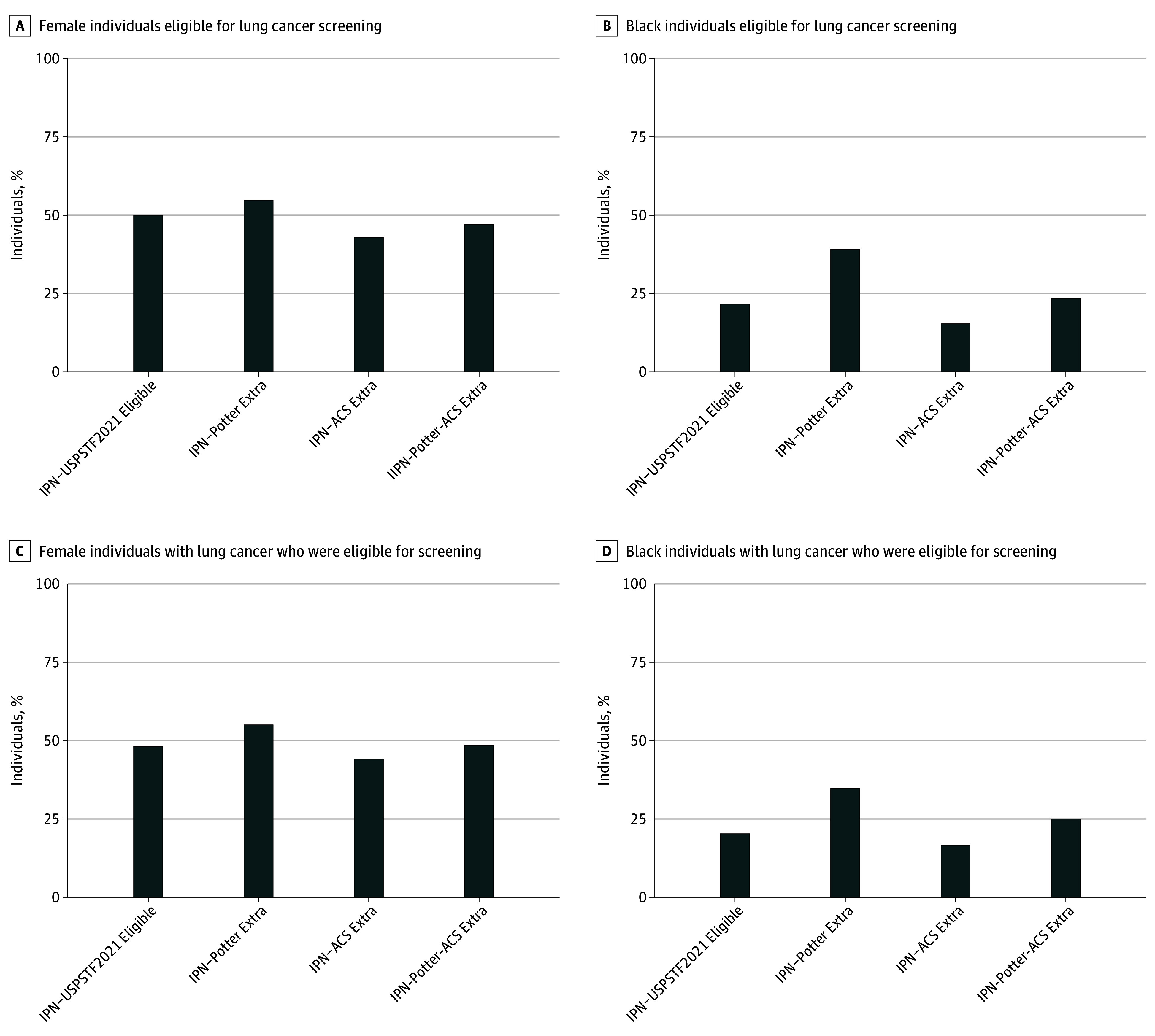
Female and Black Individuals Eligible for Lung Cancer Screening by Varying Criteria Among All Incidental Pulmonary Nodule (IPN) ACS, American Cancer Society; USPSTF, United States Preventive Services Task Force.

To better understand the impact of expanding the eligibility criteria, we examined the additional persons eligible for screening. These individuals were not eligible by USPSTF 2021 but would have been rendered eligible by each of the new criteria. Of these groups, the additional number of eligible females included 606 of 1103 (55%) with IPN-Potter Extra, 628 of 1423 (44%) with IPN-ACS Extra, and 1200 of 2467 (49%) with IPN-Potter-ACS Extra, with significant differences in the numbers of females included between the IPN-Potter Extra and IPN-USPSTF 2021 criteria (55% vs 48%; *P* < .001) (Figure 2). Furthermore, the additional eligible individuals of Black race included 382 of 1103 (35%) with IPN-Potter Extra, 237 of 1423 (17%) with IPN-ACS Extra, and 615 of 2467 (25%) with IPN-Potter-ACS Extra, with significant differences between the IPN-Potter criteria and IPN-USPSTF 2021 criteria (35% vs 20%; *P* < .001). The additional eligible persons in the highest (poorest) ADI quintile included 427 of 1103 (39%) with IPN-Potter Extra, 453 of 1423 (32%) with IPN-ACS Extra, and 847 of 2467 (34%) with IPN-Potter-ACS Extra, compared with 1491 of 3840 eligible persons (39%) from IPN-USPSTF 2021 (eFigure 2 in Supplement 1).

### Screening Eligibility in Those With Lung Cancer

Lung cancer was detected in 447 of 11 244 (4%; median [IQR] follow-up of 2.2 [1.0 to 4.0] years) of the LCS-eligible group and 270 of 1510 (18%; median [IQR] follow-up, 2.0 [1.0 to 4.0] years) of LCS-Eligible-RADS 3-4 cohorts. In the IPN-screening ineligible cohort, lung cancer was detected in 663 of 23 444 individuals (3%). When we evaluated the IPN cohort based on various eligibility criteria (USPSTF 2021, IPN-Potter, IPN-ACS, and IPN-Potter-ACS), 747 of 3840 (20%), 872 of 4905 (18%), 955 of 5263 (18%), 1051 of 6307 (17%), respectively, were diagnosed with lung cancer over a median (IQR) follow-up time ranging from 2.4 (0.9 to 4.9) years to 2.5 (1.0 to 5.1) years (eTable 1 in Supplement 1). In this cohort, 2526 individuals in the LCS group did not have documented eligibility by USPSTF 2021, and of those, 57 individuals (2%) were diagnosed with lung cancer.

In the entire IPN cohort, the number of individuals eligible for screening for every lung cancer identified was 15.8 and in those eligible by various criteria, it was 4.7 for USPSTF 2021, 5.6 for Potter criteria, 5.5 for ACS criteria, and 6.0 for Potter-ACS criteria (Figure 1). Individuals who had lung cancer had a median (IQR) age from 68 (62 to 73) years to 69 (63 to 74) years across the IPN expanded eligibility groups, with a median (IQR) of 68 (62 to 73) years in the USPSTF 2021 (eTable 2 in Supplement 1). Individuals with lung cancer in the expanded IPN groups (IPN-Potter, IPN-ACS, and IPN-Potter-ACS) were 48% to 50% female overall (459 of 955 for IPN-ACS criteria; 440 of 872 for IPN-Potter criteria; 513 of 1051 for IPN-Potter-ACS criteria) compared with 50% (370 of 747) in IPN-USPSTF 2021; 20% to 24% Black race (194 of 955 for IPN-ACS criteria; 212 of 872 for IPN-Potter criteria; 235 of 1051 for IPN-Potter-ACS criteria) vs 22% (162 of 747) in IPN-USPSTF 2021; 29% to 30% rural (284 of 955 for IPN-ACS criteria; 260 of 872 for IPN-Potter criteria; 306 of 1051 for IPN-Potter-ACS criteria) vs 31% (228 of 747) in IPN-USPSTF 2021; 38% to 39% in the highest (poorest) ADI quintile (365 of 955 for IPN-ACS criteria; 342 of 872 for IPN-Potter criteria; 402 of 1051 for IPN-Potter-ACS criteria) vs 39% (292 of 747) in IPN-USPSTF 2021 (Figure 2 and eTable 2 in Supplement 1).

The additional eligible individuals with lung cancer in IPN (IPN-Potter Extra, IPN-ACS Extra, IPN-Potter-ACS Extra) who were female included 70 of 128 (55%) in Potter criteria, 89 of 208 (43%) in ACS criteria, and 143 of 304 (47%) in Potter-ACS criteria, respectively; not significantly different between IPN-Potter Extra and IPN-USPSTF 2021 (55% vs 50% [370 of 747]; *P* = .28) (Figure 2). In terms of race, these additional eligible individuals who were Black included 50 of 128 (39%), 32 of 208 (15%), 73 of 304 (24%), respectively; with significant differences between IPN-Potter Extra and IPN-USPSTF 2021 (39% vs 22% [162 of 747]; *P* < .001). Lung cancers identified by IPN were most frequently adenocarcinoma and the clinical stage appeared similar across groups (eTable 2 in Supplement 1).

### Further Evaluation of Pack-Years and Years Smoking History

**Figure 3.**
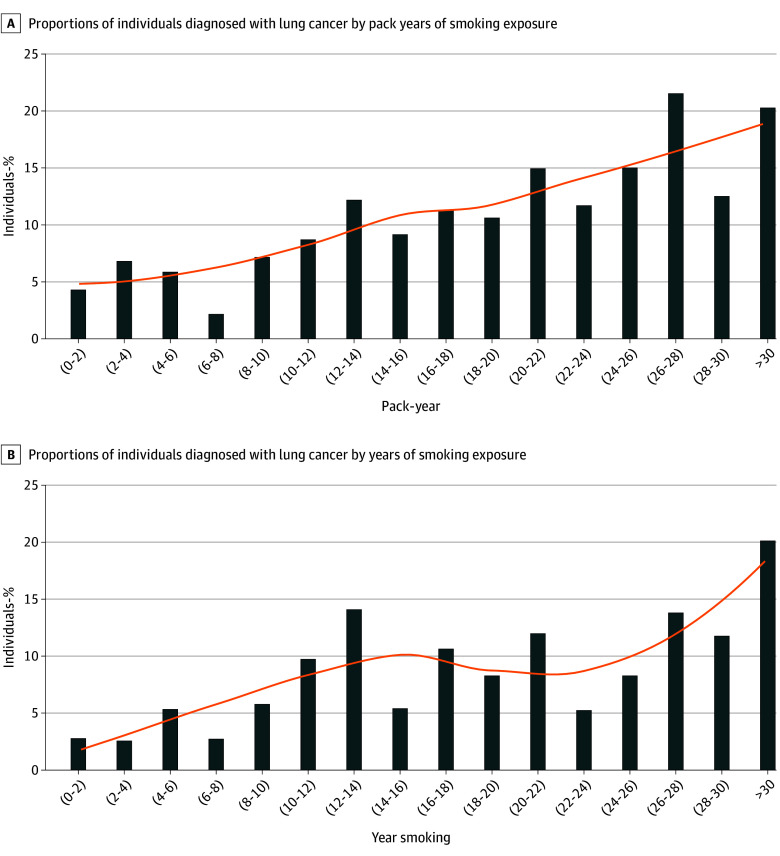
The Proportion of Individuals Diagnosed With Lung Cancer in 2-Year Increments (Patient-Years, Years Smoked) From 0 to More Than 30 (3A-B) for the Entire Detecting Early Lung Cancer Incidental Pulmonary Nodule (DELUGE) Cohort

We further evaluated potential cut-offs for pack-years and years smoked graphically (Figure 3 and eFigure 3 in Supplement 1). Visual inspection did not yield clear inflection points for the proportion with cancer, showing a more continuous upward trend. However, the figures suggest that those with 10 to 14 years or pack-years of smoking in our cohort may have an elevated probability of cancer. In our IPN cohort, including everyone older than 50 years, who smoked for 10 years or 10 pack-years, without a quit duration requirement, would yield 7993 of the 29 751 eligible (27%). Among these eligible individuals, 1251 of 7993 (16%) were diagnosed with lung cancer. Among the additional eligible individuals in this group, beyond IPN-USPSTF 2021, the median (IQR) age was 69 (61-76) years, 2006 of 4153 were female (48%) vs 2147 of 4153 were male (52%), 963 of 4153 were Black individuals (23%) vs 3113 of 4153 were White individuals (75%) vs 77 of 4153 were other race (2%).

Overall, IPN-Potter, which used years smoked rather than pack-years, would recommend screening 4905 vs 3840 people (28% increase), which yielded 872 vs 747 lung cancers identified (17% increase) over IPN-USPSTF 2021 (eFigure 1 in Supplement 1). IPN-Potter-ACS criteria would recommend screening 6307 vs 3840 people (64% increase), which yielded 1051 vs 747 lung cancers identified (41% increase) over IPN-USPSTF 2021. The final exploratory group, using 10 years or 10 pack-years would have screened 7993 vs 3840 persons (108% increase) persons (108% increase), which yielded 1251 vs 747 lung cancers identified (67% increase) over IPN-USPSTF 2021 eligible.

### Analysis of Cohort With Minimum of 2 Year Follow-Up

Of the 19 996 individuals in the IPN cohort with a minimum of 2 years of follow-up, 2658 (13%) were eligible with IPN-USPSTF 2021 criteria, 3415 (17%) with IPN-Potter, 3674 (18%) with IPN-ACS, and 4373 (22%) with IPN-Potter-ACS (eTable 3 and 4 in Supplement 1). Of these, 593 of 2658 were diagnosed with lung cancer with IPN-USPSTF 2021 (22%), 698 of 3415 with IPN-Potter (20%), 766 of 3674 with IPN-ACS (21%), and 841 of 4373 with IPN-Potter-ACS (19%) were diagnosed with lung cancer.

The individuals who were not eligible for screening by USPSTF 2021 but would have been rendered eligible for screening by each of the new criteria included 423 of 774 females (55%) with IPN-Potter Extra, 440 of 1016 females (43%) with IPN-ACS Extra, and 823 of 1715 females (48%) with the IPN-Potter-ACS Extra. The Black individuals who were not eligible for screening by the USPSTF 2021 but would have been rendered eligible by each of the new criteria included 266 of 774 (34%) by the IPN-Potter Extra, 164 of 1016 (16%) by the IPN-ACS Extra, and 421 of 1715 (25%) by the IPN-Potter-ACS Extra. The additional females with lung cancer who were eligible for screening included 56 of 107 (52%) with IPN-Potter Extra, 71 of 173 (41%) with IPN-ACS Extra, and 111 of 248 (45%) with IPN-Potter-ACS Extra. The additional Black individuals with lung cancer who were eligible for screening included 42 of 107 (39%) with IPN-Potter Extra, 27 of 173 (16%) with IPN-ACS Extra, and 59 of 248 (24%) with IPN-Potter-ACS Extra.

## Discussion

We compared currently adopted and proposed LCS eligibility criteria in a large regional early detection cohort that includes LCS and IPN programs.^12,16^ Our findings suggest that screening criteria could benefit from expanding access beyond USPSTF 2021. In this cohort study, relaxed smoking history requirements provided more frequent eligibility for LCS while maintaining reasonable diagnostic efficiency in terms of number of eligible individuals for every lung cancer identified. The shift from 20 pack-years to 20-year smoking history reduced race-based and sex-based disparities in LCS eligibility and race-based disparities in lung cancer diagnosis, while still reaching the most socially disadvantaged groups. We also found that 20-year smoking history or 20 pack-years, with no quit duration requirement, would allow for the greatest number of eligible individuals without substantial loss of efficiency.

The potential impact of a shift from 20 pack-years to 20-year smoking history has been recently demonstrated in the Southern Community Cohort Study and Black Women’s Health Study cohorts.^13^ These analyses suggested that the change would increase the proportion of individuals with lung cancer who were eligible, while eliminating race-based and sex-based disparities in eligibility. Results from our IPN cohort support the notion that the 20-year smoking criteria reduces race-based disparities in screening eligibility among those with a lung cancer diagnosis. We expanded this finding to all individuals with a lung nodule in our cohort, regardless of cancer diagnosis, where we found the 20-year smoking criteria reduced the sex-based and race-based disparity in eligibility for screening. The ACS criteria alone expanded eligibility but did not address these disparities.

In our LCS program, the USPSTF 2021 criteria currently identified 1 lung cancer for every 25.2 individuals screened. In our higher risk nodule cohort, it was 4.7 using USPSTF 2021 and increased by 28% (from 4.7 to 6.0) when we expanded the criteria to include those with 20 years of smoking history and removed the quit duration requirement. A similar increase in our screened population would result in 1 lung cancer for every 32 screened. For context, the current estimates of numbers needed to screen to find 1 cancer for breast cancer screening ranged from 337 to 1904, and estimates for colorectal cancer screening ranged from 270 to 1429.^21,22^

Risk prediction models incorporate additional risk factors besides age and smoking exposure and may be more accurate in identifying the population truly at risk for lung cancer.^23^ Programs in Canada and England use risk calculators in selecting candidates for LCS.^23,24^ Furthermore, recent work that enhances demographic models with proteomics or genomic information has shown promise for future clinical utility.^25,26^ Risk modeling approaches were rejected in the most recent Medicare Coverage Decision in 2022.^5,18,19^ Although risk models may improve the accuracy of risk assessment, they are more cumbersome, requiring additional details that may not be readily available. Furthermore, they may skew eligible populations toward older age, thereby potentially reducing the additional life-years gained from early detection. With a screening participation rate of only 5% to 20% of eligible individuals in the US, more complexity could be counterproductive at this time.^27^ Conversely, the simplicity of a 20-year smoking history with no quit duration requirement as an option may improve access by reducing 2 barriers, complexity of criteria and self-assessment recall of pack-years. Those with known history of more than 20 pack-years in less than 20 years, could be included without loss of eligibility or simplicity for others. Our data supported reducing the smoking eligibility criteria for LCS to a 20-year smoking history or 20 pack-years in individuals who are 50 to 80 years.

In exploratory analysis evaluating 10 pack-years or a 10-year smoking history, we found a large increase in both the screening eligible population and those diagnosed with lung cancer. Our data suggest there is a continuum, with the risk from cigarette smoking generally increasing with smoking years or pack-years of exposure. Thus, a clear cut-off for risk-benefit balance may be hard to fully quantify and could vary based on other risk factors, individual risk tolerance, and subpopulations with biologic factors yet unknown.^3,4,28,29,30,31^

### Limitations

This study has potential limitations, such as the study population has a predominantly Black and White racial distribution, with few individuals of Asian ancestry or Hispanic ethnicity in this Mississippi Delta cohort. Race and smoking history were self-reported and the type of cigarette smoked was not collected. Although the use of the nodule cohort to assess screening provides an opportunity to evaluate large numbers of high-risk groups relatively independent of the smoking history, conditioning on having an identified nodule limits our ability to generalize directly to the broader population and we cannot directly assess the potential impact of these various screening criteria in a population without lung nodules. It is possible that associations observed are not causal and only exist in a selected population. Therefore, we must consider this evidence in the context of other population-based studies.

## Conclusions

Our study suggests that relaxed smoking history requirements may provide better access to LCS while maintaining diagnostic efficiency. Including 20 pack-years or a 20-year smoking history with no pack-year requirement and eliminating the quit duration requirement included more females and Black individuals while maintaining reasonable efficiency and still reaching the most socially disadvantaged groups. Further expansion to a 10-year smoking history or 10 pack-years should also be explored. Our data supported expansion of the LCS eligibility criteria.

## Notes

Supplement 1. **eTable 1.** Cohort Characteristics by Screening Criteria Eligibility **eTable 2.** Lung Cancer Patient Characteristics by Lung Screening Criteria Eligibility **eTable 3.** Cohort Characteristics by USPSTF LDCT Screening Criteria Eligibility In Those With More Than 2 Years of Follow-up **eTable 4.** Lung Cancer Patients Characteristics in Different Criteria by USPSTF Screening Eligibility In Those With More Than 2 Years of Follow-Up **eFigure 1.** Numbers Eligible and With Lung Cancer by Group **eFigure 2.** Area Deprivation Index (ADI) Quartiles in Enrollees and Lung Cancer By Eligibility Group **eFigure 3.** Proportions of Females and Black Individuals Diagnosed With Lung Cancer by Pack-Years of Smoking Exposure and Years of Smoking Exposure

Supplement 2.
Data Sharing Statement
