# Supplementary material for: Reducing Smoking Requirements for Lung Screening to Address Health Disparities in a Community Cohort
Source: JAMA Netw Open. 2025 Jun 24;8(6):e2517149. doi: 10.1001/jamanetworkopen.2025.17149 (PMC12188365; doi:10.1001/jamanetworkopen.2025.17149)
Supplement: Supplement 1. — eTable 1. Cohort Characteristics by Screening Criteria Eligibility eTable 2. Lung Cancer Patient Characteristics by Lung Screening Criteria Eligibility eTable 3. Cohort Characteristics by USPSTF LDCT Screening Criteria Eligibility In Those With More Than 2 Years of Follow-up eTable 4. Lung Cancer Patients Characteristics in Different Criteria by USPSTF Screening Eligibility In Those With More Than 2 Years of Follow-Up eFigure 1. Numbers Eligible and With Lung Cancer by Group eFigure 2. Area Deprivation Index (ADI) Quartiles in Enrollees and Lung Cancer By Eligibility Group eFigure 3. Proportions of Females and Black Individuals Diagnosed With Lung Cancer by Pack-Years of Smoking Exposure and Years of Smoking Exposure [file jamanetwopen-e2517149-s001.pdf]

Supplemental Online Content

Smeltzer MP, Liao W, Goss J, et al. Reducing smoking requirements for lung screening to address health disparities in a community cohort. *JAMA Netw Open*. 2025;8(6):e2517149. doi:10.1001/jamanetworkopen.2025.17149

eTable1. Cohort Characteristics by Screening Criteria Eligibility

eTable 2. Lung Cancer Patient Characteristics by Lung Screening Criteria Eligibility

eTable 3. Cohort Characteristics by USPSTF LDCT Screening Criteria Eligibility In Those With More Than 2 Years of Follow-up

eTable 4. Lung Cancer Patients Characteristics in Different Criteria by USPSTF Screening Eligibility In Those With More Than 2 Years of Follow-Up

eFigure 1. Numbers Eligible and With Lung Cancer by Group

eFigure 2. Area Deprivation Index (ADI) Quartiles in Enrollees and Lung Cancer By Eligibility Group

eFigure 3. Proportions of Females and Black Individuals Diagnosed With Lung Cancer by Pack-Years of Smoking Exposure and Years of Smoking Exposure

This supplemental material has been provided by the authors to give readers additional information about their work.

© 2025 Smeltzer MP et al. *JAMA Network Open*.

eTable1. Cohort Characteristics by Screening Criteria Eligibility

Characteristics	LCS-Eligible<br>N = 11244	LCS-Eligible RADS 3-4<br>N = 1510	IPN-Ineligible<br>N = 23444	IPN-USPSTF2021 Eligible<br>N = 3840	IPN-Potter<br>N = 4905	IPN-ACS<br>N = 5263	IPN-Potter-ACS<br>N = 6307	IPN-Potter Extra<br>N = 1103	IPN-ACS Extra<br>N = 1423	IPN-Potter-ACS Extra<br>N = 2467
**Follow-up from the initial scan (years)**										
Missing	14(0.12)	7(0.46)	67(0.26)	28(0.73)	37(0.75)	38(0.72)	42(0.67)	9(0.82)	10(0.7)	14(0.57)
Sum	34	24	89.03	48.33	57.61	58.75	62.88	30.34	31.79	35.55
Mean (SD)	2.67(2)	2.4(2)	3.42(2.35)	3.04(2.52)	3.11(2.51)	3.13(2.52)	3.16(2.51)	3.3(2.44)	3.38(2.5)	3.34(2.47)
Median (Q1-Q3)	2.24(1-4)	1.95(1-4)	2.93(1.41-5.23)	2.43(0.89-4.93)	2.49(0.98-5)	2.51(0.96-5.12)	2.54(1.01-5.14)	2.75(1.23-5.2)	2.88(1.18-5.49)	2.77(1.18-5.4)
(Min-Max)	(0 - 10)	(0 - 8)	(0 - 9.04)	(0 - 9.03)	(0 - 9.03)	(0 - 9.03)	(0 - 9.03)	(0 - 8.85)	(0 - 8.85)	(0 - 8.85)
**Demo**										
**Lung Cancer (n, %)**	447(3.98)	270(17.88)	663(2.83)	747(19.45)	872(17.78)	955(18.15)	1051(16.66)	128(11.6)	208(14.62)	304(12.32)
**Screen Age**										
Missing	0	0	0	0	0	0	0	0	0	0
Mean (SD)	64.45(6.51)	65.69(6.55)	61.48(16.7)	65.37(7.81)	65.22(7.88)	66.88(7.94)	66.56(8.07)	64.6(8.1)	70.95(6.79)	68.41(8.12)
Median (Q1-Q3)	65(59-69)	66(61-71)	63(49-74)	66(59-72)	65(59-72)	67(61-73)	67(60-73)	65(58-71)	72(67-76)	70(62-75)
(Min-Max)	(50 - 80)	(50 - 80)	(1 - 107)	(50 - 80)	(50 - 80)	(50 - 80)	(50 - 80)	(50 - 80)	(50 - 80)	(50 - 80)
**Sex (n,%)**										
Missing	1(0.01)	0	1(0)	1(0.03)	1(0.02)	1(0.02)	1(0.02)	0	0	0
Male	5674(50.46)	781(51.72)	9891(42.19)	1992(51.86)	2462(50.19)	2787(52.95)	3259(51.67)	497(45.06)	795(55.87)	1267(51.36)
Female	5569(49.53)	729(48.28)	13552(57.81)	1847(48.1)	2442(49.79)	2475(47.03)	3047(48.31)	606(54.94)	628(44.13)	1200(48.64)
**Race (n, %)**										
Caucasian	8790(78.18)	1192(78.94)	15190(64.79)	2998(78.07)	3667(74.76)	4166(79.16)	4802(76.14)	700(63.46)	1168(82.08)	1804(73.13)
Black or African American	2197(19.54)	288(19.07)	7234(30.86)	777(20.23)	1154(23.53)	1014(19.27)	1392(22.07)	382(34.63)	237(16.65)	615(24.93)
Other/Unk/NR	257(2.29)	30(1.99)	1020(4.35)	65(1.69)	84(1.71)	83(1.58)	113(1.79)	21(1.9)	18(1.26)	48(1.95)

© 2025 Smeltzer MP et al. *JAMA Network Open*.

**Ethnicity (n, %)**										
Hispanic/Latino	50(0.44)	5(0.33)	494(2.11)	23(0.6)	26(0.53)	27(0.51)	32(0.51)	4(0.36)	4(0.28)	9(0.36)
Not Hispanic/Latino	10925(97.16)	1475(97.68)	22557(96.22)	3769(98.15)	4824(98.35)	5180(98.42)	6208(98.43)	1092(99)	1411(99.16)	2439(98.87)
Other/Unk/NR	269(2.39)	30(1.99)	393(1.68)	48(1.25)	55(1.12)	56(1.06)	67(1.06)	7(0.63)	8(0.56)	19(0.77)
**Insurance (n, %)**										
Medicare	6383(56.77)	923(61.13)	10804(46.08)	2288(59.58)	2889(58.9)	3377(64.16)	3941(62.49)	623(56.48)	1089(76.53)	1653(67.0)
Medicaid	441(3.92)	69(4.57)	708(3.02)	168(4.38)	217(4.42)	194(3.69)	238(3.77)	52(4.71)	26(1.83)	70(2.84)
Commercial	4264(37.92)	490(32.45)	9970(42.53)	1221(31.79)	1594(32.5)	1494(28.39)	1890(29.96)	384(34.81)	273(19.18)	669(27.12)
Self-Insured/Unk/NR	156(1.39)	28(1.85)	1962(8.37)	163(4.24)	205(4.18)	198(3.76)	238(3.77)	44(3.99)	35(2.46)	75(3.04)
**RUCA Code (n, %)**										
Missing	1(0.01)	0	24(0.1)	1(0.03)	1(0.02)	1(0.02)	1(0.02)	0	0	0
Metro Area	7065(62.83)	856(56.69)	17969(76.65)	2716(70.73)	3509(71.54)	3796(72.13)	4576(72.55)	824(74.71)	1080(75.9)	1860(75.4)
Rural Area	4178(37.16)	654(43.31)	5451(23.25)	1123(29.24)	1395(28.44)	1466(27.85)	1730(27.43)	279(25.29)	343(24.1)	607(24.6)
**ADI Group (n, %)**										
Missing	1(0.01)	0	25(0.11)	1(0.03)	1(0.02)	1(0.02)	1(0.02)	0	0	0
ADI Q1 (0-20%)	173(1.54)	17(1.13)	484(2.06)	51(1.33)	74(1.51)	81(1.54)	103(1.63)	23(2.09)	30(2.11)	52(2.11)
ADI Q2 (21-40%)	656(5.83)	68(4.5)	2265(9.66)	212(5.52)	262(5.34)	329(6.25)	406(6.44)	56(5.08)	117(8.22)	194(7.86)
ADI Q3 (41-60%)	2361(21)	288(19.07)	5454(23.26)	772(20.1)	980(19.98)	1135(21.57)	1343(21.29)	216(19.58)	363(25.51)	571(23.15)
ADI Q4 (61-80%)	3479(30.94)	487(32.25)	6813(29.06)	1313(34.19)	1683(34.31)	1773(33.69)	2116(33.55)	381(34.54)	460(32.33)	803(32.55)
ADI Q5 (81-100%)	4574(40.68)	650(43.05)	8403(35.84)	1491(38.83)	1905(38.84)	1944(36.94)	2338(37.08)	427(38.71)	453(31.83)	847(34.33)
**Risk**										
**Smoking Status (n, %)**										
Active	8015(71.28)	1068(70.73)	4862(20.74)	2736(71.25)	3435(70.03)	2909(55.27)	3455(54.78)	718(65.1)	173(12.16)	719(29.14)
Former	3229(28.72)	442(29.27)	5616(23.95)	1104(28.74)	1470(29.97)	2354(44.73)	2852(45.22)	385(34.9)	1250(87.84)	1748(70.86)

© 2025 Smeltzer MP et al. *JAMA Network Open*.

Never	0	0	11821(50.42)	0	0	0	0	0	0	0
Unk	0	0	1145(4.88)	0	0	0	0	0	0	0
**Pack Year (n, %*)**										
[0, 10)	0	0	4237(40.44)	0	176(3.59)	0	253(4.01)	176(15.96)	0	253(10.26)
[10, 20)	0	0	842(8.04)	0	525(10.7)	0	646(10.24)	525(47.6)	0	646(26.19)
[20, 30)	1248(11.1)	131(8.68)	340(3.24)	717(18.67)	750(15.29)	1083(20.57)	1083(17.17)	46(4.17)	366(25.72)	366(14.84)
[30, 40)	2574(22.89)	273(18.08)	265(2.53)	637(16.59)	649(13.23)	936(17.78)	936(14.84)	34(3.08)	299(21.01)	299(12.12)
>= 40	7422(66.01)	1106(73.25)	478(4.56)	2486(64.74)	2722(55.49)	3244(61.64)	3244(51.43	239(21.67)	758(53.27)	758(30.73)
Missing	0	0	4316(41.19)	0	83(1.69)	0	145(2.3)	83(7.52)	0	145(5.88)
**Quit Year(n, %*)**										
[0, 15] years	3229(100)	442(100)	1129(20.1)	1104(100)	1470(100)	1251(53.14)	1489(52.21)	385(100)	147(11.76)	385(22.03)
[16, 25) years	0	0	575(10.24)	0	0	445(18.9)	538(18.86)	0	445(35.6)	538(30.78)
>= 25 years	0	0	1687(30.04)	0	0	453(19.24)	547(19.18)	0	453(36.24)	547(31.29)
Missing	0	0	2225(39.62)	0	0	205(8.71)	278(9.75)	0	205(16.4)	278(15.9)
**COPD (n, %)**										
No	6649(59.13)	788(52.19)	20705(88.32)	1849(48.15)	2449(49.93)	2609(49.57)	3326(52.74)	621(56.3)	760(53.41)	1477(59.87)
Yes	4595(40.87)	722(47.81)	2739(11.68)	1991(51.85)	2457(50.07)	2654(50.43)	2981(47.26)	482(43.7)	663(46.59)	990(40.13)
**Comorbidity distribution**										
Mean (SD)	1.45(1.33)	1.47(1.25)	1.12(1.29)	1.83(1.42)	1.86(1.45)	1.93(1.45)	1.89(1.46)	1.94(1.52)	2.2(1.48)	1.97(1.5)
Median (Q1-Q3)	1(0 - 2)	1(1 - 2)	1(0-2)	2(1-3)	2(1-3)	2(1-3)	2(1-3)	2(1-3)	2(1-3)	2(1-3)
(Min-Max)	(0 - 9)	(0 - 7)	(0 - 9)	(0 - 8)	(0 - 8)	(0 - 8)	(0 - 8)	(0 - 8)	(0 - 8)	(0 - 8)
**Charlson comorbidity (n, %)**										
0	2941(26.16)	341(22.58)	9660(41.2)	638(16.61)	824(16.8)	784(14.89)	1034(16.39)	190(17.23)	146(10.26)	396(16.05)
1	5224(46.46)	759(50.26)	8389(35.78)	1766(45.99)	2135(43.53)	2245(42.66)	2674(42.4)	391(35.45)	479(33.66)	908(36.81)

© 2025 Smeltzer MP et al. *JAMA Network Open*.

2	3079(27.38)	410(27.15)	5395(23.01)	1436(37.4)	1946(39.67)	2234(42.45)	2599(41.21)	522(47.33)	798(56.08)	1163(47.14)
**Prior history of cancer (n, %)**										
No	9156(81.43)	1214(80.4)	18729(79.89)	2980(77.6)	3552(72.42)	3665(69.64)	4441(70.41)	603(54.67)	685(48.14)	1461(59.22)
Yes	2088(18.57)	296(19.6)	4715(20.11)	860(22.4)	1353(27.58)	1598(30.36)	1866(29.59)	500(45.33)	738(51.86)	1006(40.78)
**Prior LUNG cancer (n, %)**										
No	2088(18.57)	296(19.6)	4321(18.43)	860(22.4)	997(20.33)	1204(22.88)	1425(22.59)	144(13.06)	344(24.17)	565(22.9)
Yes	0	0	394(1.68)	0	356(7.26)	394(7.49)	441(6.99)	356(32.28)	394(27.69)	441(17.88)
**Family history of cancer (n, %)**										
No	3004(26.72)	421(27.88)	7361(31.4)	1149(29.92)	1464(29.85)	1577(29.96)	1903(30.18)	325(29.47)	428(30.08)	754(30.56)
Yes	5797(51.56)	776(51.39)	8051(34.34)	2004(52.19)	2568(52.35)	2849(54.13)	3360(53.27)	585(53.04)	845(59.38)	1356(54.979)
Status Unknown	2443(21.73)	313(20.73)	8032(34.26)	687(17.89)	873(17.79)	837(15.9)	1044(16.55)	193(17.5)	150(10.54)	357(14.47)
**Family LUNG cancer (n, %)**										
No	4442(39.51)	577(38.21)	6595(28.13)	1489(38.77)	1900(38.74)	2115(40.19)	2515(39.88)	431(39.08)	626(43.99)	1026(41.59)
Yes	1355(12.05)	199(13.18)	1456(6.21)	515(13.41)	668(13.62)	734(13.95)	845(13.4)	154(13.96)	219(15.39)	330(13.38)

© 2025 Smeltzer MP et al. *JAMA Network Open*.

Characteristics	LCS-Eligible N = 447	LCS-Eligible RADS3-4 N = 270	IPN-Ineligible N = 663	IPN-USPSTF2021 Eligible N = 747	IPN-Potter N = 872	IPN-ACS N = 955	IPN-Potter-ACS N = 1051	IPN-Potter Extra N = 128	IPN-ACS Extra N = 208	IPN-Potter-ACS Extra N = 304
**Demo**										
**Screen Age**										
Mean (SD)	67.46(5.85)	67.85(5.86)	71.87(12.44)	67.16(7.2)	67.09(7.35)	68.19(7.29)	68.1(7.43)	66.59(8.16)	71.89(6.34)	70.4(7.48)
Median (Q1-Q3)	68(63-72)	68(64-72.75)	74(64-82)	68(62-73)	68(62-73)	69(63-74)	69(63-74)	67(60-73.25)	73(68-77)	72(65.75-76)
(Min-Max)	(50 - 79)	(50 - 79)	(33 - 100)	(50 - 80)	(50 - 94)	(50 - 80)	(50 - 80)	(50 - 80)	(50 - 80)	(50 - 80)
**Sex (n,%)**										
Missing	0	0	0	1(0.13)	1(0.11)	1(0.1)	1(0.1)	0	0	0
Male	213(47.65)	125(46.3)	321(48.42)	376(50.33)	431(49.43)	495(51.83)	537(51.09)	58(45.31)	119(57.21)	161(52.96)
Female	234(52.35)	145(53.7)	342(51.58)	370(49.53)	440(50.46)	459(48.06)	513(48.81)	70(54.69)	89(42.79)	143(47.04)
**Race (n, %)**										
Caucasian	367(82.1)	214(79.26)	444(66.97)	579(77.51)	653(74.89)	754(78.95)	808(76.88)	77(60.16)	175(84.13)	229(75.33)
Black or African American	74(16.55)	51(18.89)	198(29.86)	162(21.69)	212(24.31)	194(20.31)	235(22.36)	50(39.06)	32(15.38)	73(24.01)
Other/Unk/NR	6(1.34)	5(1.85)	21(3.17)	6(0.8)	7(0.8)	7(0.73)	8(0.76)	1(0.78)	1(0.48)	2(0.66)
**Ethnicity (n, %)**										
Hispanic/Latino	1(0.22)	0	7(1.06)	3(0.4)	3(0.34)	4(0.42)	4(0.38)	0	1(0.48)	1(0.33)
Not Hispanic/Latino	436(97.54)	263(97.41)	651(98.19)	736(98.53)	861(98.74)	942(98.64)	1038(98.76)	128(100)	206(99.04)	302(99.34)
Other/Unk/NR	10(2.24)	7(2.59)	5(0.75)	8(1.07)	8(0.92)	9(0.94)	9(0.86)	0	1(0.48)	1(0.33)
**Insurance (n, %)**										
Medicare	308(68.9)	186(68.89)	464(69.98)	493(66)	585(67.09)	667(69.84)	730(69.46)	93(72.66)	174(83.65)	237(77.96)
Medicaid	16(3.58)	11(4.07)	16(2.41)	28(3.75)	30(3.44)	30(3.14)	32(3.04)	3(2.34)	2(0.96)	4(1.32)
Commercial	120(26.85)	71(26.3)	148(22.32)	193(25.84)	221(25.34)	222(23.25)	250(23.79)	29(22.66)	29(13.94)	57(18.75)
Self-Insured/Unk/NR	3(0.67)	2(0.74)	35(5.28)	33(4.42)	36(4.13)	36(3.77)	39(3.71)	3(2.34)	3(1.44)	6(1.97)
**RUCA Code (n, %)**										
Missing	0	0	0	0	0	0	0	0	0	0
Metro Area	295(66)	177(65.56)	513(77.38)	519(69.48)	612(70.18)	671(70.26)	745(70.88)	96(75)	152(73.08)	226(74.34)
Rural Area	152(34)	93(34.44)	150(22.62)	228(30.52)	260(29.82)	284(29.74)	306(29.12)	32(25)	56(26.92)	78(25.66)
**ADI Group (n, %)**										
Missing	0	0	0	0	0	0	0	0	0	0
ADI Q1 (0-20%)	4(0.89)	4(1.48)	11(1.66)	9(1.2)	14(1.61)	12(1.26)	16(1.52)	5(3.91)	3(1.44)	7(2.3)
ADI Q2 (21-40%)	19(4.25)	13(4.81)	72(10.86)	36(4.82)	40(4.59)	62(6.49)	66(6.28)	5(3.91)	26(12.5)	30(9.87)

© 2025 Smeltzer MP et al. *JAMA Network Open*.

ADI Q3 (41-60%)	102(22.82)	59(21.85)	166(25.04)	154(20.62)	180(20.64)	200(20.94)	223(21.22)	27(21.09)	46(22.12)	69(22.7)
ADI Q4 (61-80%)	133(29.75)	78(28.89)	188(28.36)	256(34.27)	296(33.94)	316(33.09)	344(32.73)	40(31.25)	60(28.85)	88(28.95)
ADI Q5 (81-100%)	189(42.28)	116(42.96)	226(34.09)	292(39.09)	342(39.22)	365(38.22)	402(38.25)	51(39.84)	73(35.1)	110(36.18)
**Risk**										
**Smoking Status (n, %)**										
Active	331(74.05)	193(71.48)	215(32.43)	549(73.49)	631(72.36)	572(59.9)	632(60.13)	83(64.84)	23(11.06)	83(27.3)
Former	116(25.95)	77(28.52)	274(41.33)	198(26.51)	241(27.64)	383(40.1)	419(39.87)	45(35.16)	185(88.94)	221(72.7)
Never	0	0	162(24.43)	0	0	0	0	0	0	0
Unk	0	0	12(1.81)	0	0	0	0	0	0	0
**Pack Year (n, %*)**										
[0, 10)	0	0	157(32.11)	0	12(1.38)	0	15(1.43)	12(9.38)	0	15(4.93)
[10, 20)	0	0	54(11.04)	0	59(6.77)	0	68(6.47)	59(46.09)	0	68(22.37)
[20, 30)	36(8.05)	27(10)	23(4.7)	94(12.58)	104(11.93)	145(15.18)	145(13.8)	11(8.59)	51(24.52)	51(16.78)
[30, 40)	40(8.95)	28(10.37)	27(5.52)	77(10.31)	79(9.06)	115(12.04)	115(10.94)	4(3.12)	38(18.27)	38(12.5)
>= 40	371(83)	215(79.63)	82(16.77)	576(77.11)	611(70.07)	695(72.77)	695(66.13)	35(27.34)	119(57.21)	119(39.14)
Missing	0	0	146(29.86)	0	7(0.8)	0	13(1.24)	7(5.47)	0	13(4.28)
**Quit Year(n, %*)**										
[0, 15] years	116(100)	77(100)	56(20.44)	198(100)	241(100)	225(58.75)	243(58)	45(100)	27(14.59)	45(20.36)
[16, 25) years	0	0	41(14.96)	0	0	77(20.1)	81(19.33)	0	77(41.62)	81(36.65)
>= 25 years	0	0	117(42.7)	0	0	70(18.28)	81(19.33)	0	70(37.84)	81(36.65)
Missing	0	0	60(21.9)	0	0	11(2.87)	14(3.34)	0	11(5.95)	14(6.33)
**COPD (n, %)**										
No	202(45.19)	130(48.15)	490(73.91)	344(46.05)	405(46.44)	458(47.96)	518(49.29)	61(47.66)	114(54.81)	174(57.24)
Yes	245(54.81)	140(51.85)	173(26.09)	403(53.95)	467(53.56)	497(52.04)	533(50.71)	67(52.34)	94(45.19)	130(42.76)
**Comorbidity Distribution**										
Mean (SD)	1.69(1.37)	1.58(1.29)	1.37(1.33)	1.71(1.35)	1.75(1.37)	1.78(1.37)	1.76(1.37)	1.94(1.49)	2.01(1.4)	1.87(1.41)
Median (Q1-Q3)	1(1-2)	1(1-2)	1(0-2)	1(1-2)	2(1-2)	2(1-3)	2(1-3)	2(1-3)	2(1-3)	2(1-3)
(Min-Max)	(0 - 7)	(0 - 7)	(0 - 7)	(0 - 7)	(0 - 7)	(0 - 7)	(0 - 7)	(0 - 6)	(0 - 6)	(0 - 6)
**Charlson comorbidity (n, %)**										
0	78(17.45)	53(19.63)	202(30.47)	138(18.47)	157(18)	162(16.96)	185(17.6)	19(14.84)	24(11.54)	47(15.46)
1	223(49.89)	139(51.48)	267(40.27)	358(47.93)	404(46.33)	434(45.45)	477(45.39)	48(37.5)	76(36.54)	119(39.14)
2	146(32.66)	78(28.89)	194(29.26)	251(33.6)	311(35.67)	359(37.59)	389(37.01)	61(47.66)	108(51.92)	138(45.39)

© 2025 Smeltzer MP et al. *JAMA Network Open*.

**Prior history of Cancer (n, %)**										
No	341(76.29)	216(80)	482(72.7)	578(77.38)	638(73.17)	681(71.31)	756(71.93)	63(49.22)	103(49.52)	178(58.55)
Yes	106(23.71)	54(20)	181(27.3)	169(22.62)	234(26.83)	274(28.69)	295(28.07)	65(50.78)	105(50.48)	126(41.45)
**Prior LUNG cancer (n, %)**										
No	106(23.71)	54(20)	148(22.32)	169(22.62)	183(20.99)	214(22.41)	233(22.17)	14(10.94)	45(21.63)	64(21.05)
Yes	0	0	33(4.98)	0	51(5.85)	60(6.28)	62(5.9)	51(39.84)	60(28.85)	62(20.39)
**Family history of cancer (n, %)**										
No	128(28.64)	82(30.37)	210(31.67)	180(24.1)	209(23.97)	239(25.03)	261(24.83)	29(22.66)	59(28.37)	81(26.64)
Yes	264(59.06)	149(55.19)	293(44.19)	488(65.33)	562(64.45)	618(64.71)	673(64.03)	77(60.16)	130(62.5)	185(60.86)
Status Unknown	55(12.3)	39(14.44)	160(24.13)	79(10.58)	101(11.58)	98(10.26)	117(11.13)	22(17.19)	19(9.13)	38(12.5)
**Family LUNG cancer (n, %)**										
No	171(38.26)	100(37.04)	221(33.33)	308(41.23)	356(40.83)	393(41.15)	433(41.2)	51(39.84)	85(40.87)	125(41.12)
Yes	93(20.81)	49(18.15)	72(10.86)	180(24.1)	206(23.62)	225(23.56)	240(22.84)	26(20.31)	45(21.63)	60(19.74)
**Cancer**										
**Histology (n, %)**										
Adenocarcinoma	177(39.6)	130(48.15)	348(52.49)	320(42.84)	382(43.81)	432(45.24)	480(45.67)	64(50)	112(53.85)	160(52.63)
Squamous cell ca	158(35.35)	87(32.22)	137(20.66)	216(28.92)	246(28.21)	268(28.06)	291(27.69)	31(24.22)	52(25)	75(24.67)
Adenosquamous ca	1(0.22)	1(0.37)	4(0.6)	7(0.94)	8(0.92)	9(0.94)	10(0.95)	1(0.78)	2(0.96)	3(0.99)
Large cell	12(2.68)	8(2.96)	13(1.96)	20(2.68)	23(2.64)	23(2.41)	25(2.38)	3(2.34)	3(1.44)	5(1.64)
Small cell Lung ca	69(15.44)	30(11.11)	65(9.8)	97(12.99)	116(13.3)	117(12.25)	133(12.65)	19(14.84)	20(9.62)	36(11.84)
Other	29(6.49)	14(5.19)	88(13.27)	86(11.51)	96(11.01)	104(10.89)	110(10.47)	10(7.81)	18(8.65)	24(7.89)
Unk/NR	1(0.22)	0	8(1.21)	1(0.13)	1(0.11)	2(0.21)	2(0.19)	0	1(0.48)	1(0.33)
**Primary Tumor Size (cm)**										
Missing	30(6.71)	14(5.19)	65(9.8)	40(5.35)	48(5.5)	58(6.07)	68(6.47)	9(7.03)	18(8.65)	28(9.21)
Mean (SD)	2.64(1.94)	2.66(1.9)	3.78(2.55)	3.6(3.92)	3.57(3.77)	3.48(3.62)	3.49(3.54)	3.39(2.63)	3.06(2.1)	3.19(2.26)
Median (Q1-Q3)	2.1(1.3-3.1)	2.15(1.4-3.23)	3(1.9-5)	2.6(1.6-4.5)	2.5(1.6-4.53)	2.5(1.6-4.5)	2.5(1.6-4.5)	2.4(1.6-4.75)	2.25(1.6-4)	2.4(1.6-4.1)
(Min, Max)	(0.3 - 13.5)	(0.3 - 13.5)	(0.5 - 16.1)	(0.4 - 80)	(0.4 - 80)	(0.4 - 80)	(0.4 - 80)	(0.6 - 14.5)	(0.6 - 10)	(0.6 - 14.5)
**Clinical Stage (n, %)**										
Stage I	248(55.48)	151(55.93)	253(38.16)	311(41.63)	362(41.51)	416(43.56)	451(42.91)	51(39.84)	105(50.48)	140(46.05)
Stage II	30(6.71)	21(7.78)	52(7.84)	53(7.1)	67(7.68)	76(7.96)	84(7.99)	14(10.94)	23(11.06)	31(10.2)
Stage III	76(17)	47(17.41)	124(18.7)	162(21.69)	187(21.44)	197(20.63)	215(20.46)	26(20.31)	35(16.83)	53(17.43)

© 2025 Smeltzer MP et al. *JAMA Network Open*.

Stage IV	75(16.78)	40(14.81)	206(31.07)	204(27.31)	235(26.95)	240(25.13)	268(25.5)	32(25)	36(17.31)	64(21.05)
Unk/NR	18(4.03)	11(4.07)	28(4.22)	17(2.28)	21(2.41)	26(2.72)	33(3.14)	5(3.91)	9(4.33)	16(5.26)
**Clinical T Category (n, %)**										
cTX, cT0, cTis	7(1.57)	2(0.74)	14(2.11)	10(1.34)	13(1.49)	17(1.78)	19(1.81)	3(2.34)	7(3.37)	9(2.96)
cT1	284(63.53)	170(62.96)	271(40.87)	377(50.47)	438(50.23)	492(51.52)	533(50.71)	62(48.44)	115(55.29)	156(51.32)
cT2	64(14.32)	45(16.67)	129(19.46)	119(15.93)	132(15.14)	145(15.18)	157(14.94)	13(10.16)	26(12.5)	38(12.5)
cT3	35(7.83)	21(7.78)	76(11.46)	86(11.51)	103(11.81)	110(11.52)	122(11.61)	17(13.28)	24(11.54)	36(11.84)
cT4	40(8.95)	22(8.15)	144(21.72)	139(18.61)	167(19.15)	166(17.38)	189(17.98)	29(22.66)	27(12.98)	50(16.45)
Unk/NR	17(3.8)	10(3.7)	29(4.37)	16(2.14)	19(2.18)	25(2.62)	31(2.95)	4(3.12)	9(4.33)	15(4.93)
**Clinical N Category (n, %)**										
cN0	291(65.1)	181(67.04)	402(60.63)	440(58.9)	520(59.63)	582(60.94)	641(60.99)	81(63.28)	142(68.27)	201(66.12)
cN1	26(5.82)	16(5.93)	23(3.47)	45(6.02)	50(5.73)	51(5.34)	55(5.23)	5(3.91)	6(2.88)	10(3.29)
cN2	68(15.21)	37(13.7)	123(18.55)	160(21.42)	182(20.87)	191(20)	205(19.51)	22(17.19)	31(14.9)	45(14.8)
cN3	45(10.07)	26(9.63)	87(13.12)	86(11.51)	101(11.58)	106(11.1)	119(11.32)	16(12.5)	20(9.62)	33(10.86)
Unk/NR	17(3.8)	10(3.7)	28(4.22)	16(2.14)	19(2.18)	25(2.62)	31(2.95)	4(3.12)	9(4.33)	15(4.93)
**Clinical M Category (n, %)**										
cM0	354(79.19)	219(81.11)	418(63.05)	522(69.88)	612(70.18)	685(71.73)	746(70.98)	91(71.09)	163(78.37)	224(73.68)
cM1a	14(3.13)	9(3.33)	68(10.26)	39(5.22)	44(5.05)	47(4.92)	51(4.85)	5(3.91)	8(3.85)	12(3.95)
cM1b	20(4.47)	13(4.81)	47(7.09)	49(6.56)	58(6.65)	53(5.55)	63(5.99)	10(7.81)	4(1.92)	14(4.61)
cM1c	42(9.4)	19(7.04)	102(15.38)	121(16.2)	139(15.94)	145(15.18)	160(15.22)	18(14.06)	24(11.54)	39(12.83)
Unk/NR	17(3.8)	10(3.7)	28(4.22)	16(2.14)	19(2.18)	25(2.62)	31(2.95)	4(3.12)	9(4.33)	15(4.93)

© 2025 Smeltzer MP et al. *JAMA Network Open*.

eTable 3. Cohort Characteristics by USPSTF LDCT Screening Criteria Eligibility In those with >2 Years of Follow-up

Characteristics	LCS-Eligible<br>N = 6252	LCS-Eligible RADS 3-4<br>N = 885	IPN-Ineligible<br>N = 15623	IPN-USPSTF2021 Eligible<br>N = 2658	IPN-Potter<br>N = 3415	IPN-ACS<br>N = 3674	IPN-Potter-ACS<br>N = 4373	IPN-Potter Extra<br>N = 774	IPN-ACS Extra<br>N = 1016	IPN-Potter-ACS Extra<br>N = 1715
**Follow-up from the initial scan (years)**										
Missing	13(0.21)	6(0.68)	49(0.31)	24(0.9)	32(0.94)	34(0.93)	37(0.85)	8(1.03)	10(0.98)	13(0.76)
Sum	39	28	76	49	57	59	63	34	36	39
Mean (SD)	3.94(2)	3.42(2)	4.62(2)	3.96(3)	4.03(2)	4.06(2)	4.11(2)	4.26(2)	4.33(2)	4.35(2)
Median (Q1-Q3)	3.66(3-5)	3.29(2-5)	4.43(3-6)	3.62(2-6)	3.73(2-6)	3.83(2-6)	3.98(2-6)	4.13(3-6)	4.45(3-6)	4.42(3-6)
(Min-Max)	(0 - 10)	(0 - 8)	(0 - 9)	(0 - 9)	(0 - 9)	(0 - 9)	(0 - 9)	(0 - 9)	(0 - 9)	(0 - 9)
**Demo**										
**Lung Cancer (n, %)**	342(5.47)	181(20.45)	491(3.14)	593(22.31)	698(20.44)	766(20.85)	841(19.23)	107(13.82)	173(17.03)	248(14.46)
**Screen Age**										
Missing	0	0	0	0	0	0	0	0	0	0
Mean (SD)	64.91(6.07)	66.16(6.05)	60.93(16.77)	65.24(7.81)	65.14(7.92)	66.72(7.94)	66.4(8.07)	64.79(8.27)	70.59(6.91)	68.21(8.13)
Median (Q1-Q3)	65(60-69)	67(61-71)	62(49-74)	66(59-71)	65(59-71)	67(61-73)	67(60-73)	65(58-72)	72(66-76)	69(62-75)
(Min-Max)	(50 - 80)	(50 - 80)	(1 - 107)	(50 - 80)	(50 - 80)	(50 - 80)	(50 - 80)	(50 - 80)	(50 - 80)	(50 - 80)
**Sex (n,%)**										
Missing	0	0	0	0	0	0	0	0	0	0
Male	3143(50.27)	451(50.96)	6488(41.53)	1364(51.32)	1702(49.84)	1940(52.8)	2256(51.59)	351(45.35)	576(56.69)	892(52.01)
Female	3109(49.73)	434(49.04)	9135(58.47)	1294(48.68)	1713(50.16)	1734(47.2)	2117(48.41)	423(54.65)	440(43.31)	823(47.99)
**Race (n, %)**										
Caucasian	5044(80.68)	714(80.68)	10084(64.55)	2079(78.22)	2557(74.88)	2921(79.5)	3344(76.47)	493(63.7)	842(82.87)	1265(73.76)
Black or African American	1123(17.96)	161(18.19)	4893(31.32)	537(20.2)	801(23.46)	701(19.08)	958(21.91)	266(34.37)	164(16.14)	421(24.55)
Other/Unk/NR	85(1.36)	10(1.13)	646(4.13)	42(1.58)	57(1.67)	52(1.42)	71(1.62)	15(1.94)	10(0.98)	29(1.69)
**Ethnicity (n, %)**										
Hispanic/Latino	25(0.4)	3(0.34)	294(1.88)	13(0.49)	15(0.44)	16(0.44)	19(0.43)	2(0.26)	3(0.3)	6(0.35)
Not Hispanic/Latino	6109(97.71)	869(98.19)	15126(96.82)	2627(98.83)	3379(98.95)	3638(99.02)	4329(98.99)	769(99.35)	1011(99.51)	1702(99.24)
Other/Unk/NR	118(1.89)	13(1.47)	203(1.3)	18(0.68)	21(0.61)	20(0.54)	25(0.57)	3(0.39)	2(0.2)	7(0.41)

© 2025 Smeltzer MP et al. *JAMA Network Open*.

**Insurance (n, %)**										
Medicare	3790(60.62)	573(64.75)	7134(45.66)	1633(61.44)	2075(60.76)	2419(65.84)	2806(64.17)	454(58.66)	786(77.36)	1173(68.4)
Medicaid	198(3.17)	34(3.84)	455(2.91)	100(3.76)	133(3.89)	116(3.16)	143(3.27)	35(4.52)	16(1.57)	43(2.51)
Commercial	2180(34.87)	264(29.83)	6587(42.16)	800(30.1)	1049(30.72)	986(26.84)	1241(28.38)	252(32.56)	186(18.31)	441(25.71)
Self-Insured/Unk/NR	84(1.34)	14(1.58)	1447(9.26)	125(4.7)	158(4.63)	153(4.16)	183(4.18)	33(4.26)	28(2.76)	58(3.38)
**RUCA Code (n, %)**										
Missing	1(0.02)	0	19(0.12)	0	0	0	0	0	0	0
Metro Area	4079(65.24)	547(61.81)	12651(80.98)	1973(74.23)	2566(75.14)	2782(75.72)	3334(76.24)	607(78.42)	809(79.63)	1361(79.36)
Rural Area	2172(34.74)	338(38.19)	2953(18.9)	685(25.77)	849(24.86)	892(24.28)	1039(23.76)	167(21.58)	207(20.37)	354(20.64)
**ADI Group (n, %)**										
Missing	1(0.02)	0	20(0.13)	0	0	0	0	0	0	0
ADI Q1 (0-20%)	89(1.42)	10(1.13)	322(2.06)	38(1.43)	57(1.67)	61(1.66)	79(1.81)	19(2.45)	23(2.26)	41(2.39)
ADI Q2 (21-40%)	395(6.32)	48(5.42)	1622(10.38)	146(5.49)	184(5.39)	242(6.59)	298(6.81)	41(5.3)	96(9.45)	152(8.86)
ADI Q3 (41-60%)	1436(22.97)	176(19.89)	3817(24.43)	550(20.69)	701(20.53)	823(22.4)	961(21.98)	154(19.9)	273(26.87)	411(23.97)
ADI Q4 (61-80%)	1944(31.09)	296(33.45)	4586(29.35)	955(35.93)	1232(36.08)	1295(35.25)	1537(35.15)	284(36.69)	340(33.46)	582(33.94)
ADI Q5 (81-100%)	2387(38.18)	355(40.11)	5256(33.64)	969(36.46)	1241(36.34)	1253(34.1)	1498(34.26)	276(35.66)	284(27.95)	529(30.85)
**Risk**										
**Smoking Status (n, %)**										
Active	4388(70.19)	614(69.38)	3272(20.94)	1915(72.05)	2399(70.25)	2041(55.55)	2410(55.11)	495(63.95)	126(12.4)	495(28.86)
Former	1864(29.81)	271(30.62)	3618(23.16)	743(27.95)	1016(29.75)	1633(44.45)	1963(44.89)	279(36.05)	890(87.6)	1220(71.14)
Never	0	0	7842(50.2)	0	0	0	0	0	0	0
Unk	0	0	891(5.7)	0	0	0	0	0	0	0
**Pack Year (n, %*)**										
[0, 10)	0	0	1310(19.01)	0	92(2.69)	0	119(2.72)	92(11.89)	0	119(6.94)
[10, 20)	0	0	559(8.11)	0	357(10.45)	0	435(9.95)	357(46.12)	0	435(25.36)
[20, 30)	303(4.85)	39(4.41)	234(3.4)	468(17.61)	496(14.52)	725(19.73)	725(16.58)	34(4.39)	257(25.3)	257(14.99)

© 2025 Smeltzer MP et al. *JAMA Network Open*.

[30, 40)	1394(22.3)	150(16.95)	173(2.51)	428(16.1)	443(12.97)	641(17.45)	641(14.66)	25(3.23)	213(20.96)	213(12.42)
>= 40	4555(72.86)	696(78.64)	308(4.47)	1762(66.29)	1944(56.93)	2308(62.82)	2308(52.78)	183(23.64)	546(53.74)	546(31.84)
Missing	0	0	4306(62.5)	0	83(2.43)	0	145(3.32)	83(10.72)	0	145(8.45)
**Quit Year(n, %*)**										
[0, 15] years	1864(100)	271(100)	694(19.18)	743(100)	1016(100)	859(52.6)	1022(52.06)	279(100)	116(13.03)	279(22.87)
[16, 25) years	0	0	369(10.2)	0	0	296(18.13)	349(17.78)	0	296(33.26)	349(28.61)
>= 25 years	0	0	1077(29.77)	0	0	302(18.49)	356(18.14)	0	302(33.93)	356(29.18)
Missing	0	0	1478(40.85)	0	0	176(10.78)	236(12.02)	0	176(19.78)	236(19.34)
**COPD (n, %)**										
No	3470(55.5)	433(48.93)	13824(88.48)	1273(47.89)	1691(49.52)	1805(49.13)	2290(52.37)	428(55.3)	532(52.36)	1017(59.3)
Yes	2782(44.5)	452(51.07)	1799(11.52)	1385(52.11)	1724(50.48)	1869(50.87)	2083(47.63)	346(44.7)	484(47.64)	698(40.7)
**Comorbidity distribution**										
Mean (SD)	1.41(1.29)	1.46(1.25)	1.07(1.25)	1.83(1.41)	1.86(1.44)	1.91(1.44)	1.87(1.44)	1.96(1.51)	2.15(1.47)	1.94(1.49)
Median (Q1-Q3)	1(0-2)	1(1-2)	1(0-2)	2(1-3)	2(1-3)	2(1-3)	2(1-3)	2(1-3)	2(1-3)	2(1-3)
(Min-Max)	(0 - 9)	(0 - 6)	(0 - 9)	(0 - 8)	(0 - 8)	(0 - 8)	(0 - 8)	(0 - 8)	(0 - 8)	(0 - 8)
**Charlson comorbidity (n, %)**										
0	1625(25.99)	197(22.26)	6633(42.46)	440(16.55)	561(16.43)	545(14.83)	709(16.21)	124(16.02)	105(10.33)	269(15.69)
1	3011(48.16)	457(51.64)	5637(36.08)	1229(46.24)	1503(44.01)	1593(43.36)	1898(43.4)	282(36.43)	364(35.83)	669(39.01)
2	1616(25.85)	231(26.1)	3353(21.46)	989(37.21)	1351(39.56)	1536(41.81)	1766(40.38)	368(47.55)	547(53.84)	777(45.31)
**Prior history of cancer (n, %)**										
No	5074(81.16)	714(80.68)	12498(80)	2057(77.39)	2446(71.63)	2539(69.11)	3055(69.86)	403(52.07)	482(47.44)	998(58.19)
Yes	1178(18.84)	171(19.32)	3125(20)	601(22.61)	969(28.37)	1135(30.89)	1318(30.14)	371(47.93)	534(52.56)	717(41.81)
**Prior LUNG cancer (n, %)**										
No	1178(18.84)	171(19.32)	2841(18.18)	601(22.61)	701(20.53)	834(22.7)	984(22.5)	103(13.31)	233(22.93)	383(22.33)
Yes	0	0	284(1.82)	0	268(7.85)	301(8.19)	334(7.64)	268(34.63)	301(29.63)	334(19.48)

© 2025 Smeltzer MP et al. *JAMA Network Open*.

**Family history of cancer (n, %)**										
No	1598(25.56)	221(24.97)	4771(30.54)	762(28.67)	974(28.52)	1065(28.99)	1267(28.97)	216(27.91)	303(29.82)	505(29.45)
Yes	3184(50.93)	474(53.56)	5305(33.96)	1420(53.42)	1832(53.65)	2020(54.98)	2374(54.29)	423(54.65)	600(59.06)	954(55.63)
Status Unknown	1470(23.51)	190(21.47)	5547(35.51)	476(17.91)	609(17.83)	589(16.03)	732(16.74)	135(17.44)	113(11.12)	256(14.93)
**Family LUNG cancer (n, %)**										
No	2363(37.8)	337(38.08)	4294(27.49)	1045(39.32)	1339(39.21)	1485(40.42)	1756(40.16)	304(39.28)	440(43.31)	711(41.46)
Yes	821(13.13)	137(15.48)	1011(6.47)	375(14.11)	493(14.44)	535(14.56)	618(14.13)	119(15.37)	160(15.75)	243(14.17)

© 2025 Smeltzer MP et al. *JAMA Network Open*.

eTable 4. Lung Cancer Patients Characteristics in Different Criteria by USPSTF Screening Eligibility In those with >2 Years of Follow-up

Characteristics	LCS-Eligible N = 342	LCS-Eligible RADS3-4 N = 181	IPN-Ineligible N = 491	IPN-USPSTF2021 Eligible N = 593	IPN-Potter N = 698	IPN-ACS N = 766	IPN-Potter-ACS N = 841	IPN-Potter Extra N = 107	IPN-ACS Extra N = 173	IPN-Potter-ACS Extra N = 248
**Demo**										
**Screen Age**										
Mean (SD)	67.35(5.75)	67.81(5.71)	72.24(12.31)	67.21(7.17)	67.11(7.36)	68.23(7.21)	68.09(7.39)	66.48(8.32)	71.72(6.21)	70.2(7.5)
Median (Q1-Q3)	68(63-71.75)	68(64-72)	74(64.5-82)	68(62-73)	68(62-73)	69(63-74)	69(63-74)	67(59.5-74)	73(68-77)	72(65-76)
(Min-Max)	(53 - 79)	(55 - 79)	(34 - 100)	(50 - 80)	(50 - 80)	(50 - 80)	(50 - 80)	(50 - 80)	(50 - 80)	(50 - 80)
**Sex (n,%)**										
Missing	0	0	0	0	0	0	0	0	0	0
Male	165(48.25)	88(48.62)	230(46.84)	292(49.24)	341(48.85)	394(51.44)	429(51.01)	51(47.66)	102(58.96)	137(55.24)
Female	177(51.75)	93(51.38)	261(53.16)	301(50.76)	357(51.15)	372(48.56)	412(48.99)	56(52.34)	71(41.04)	111(44.76)
**Race (n, %)**										
Caucasian	295(86.26)	154(85.08)	332(67.62)	462(77.91)	524(75.07)	608(79.37)	650(77.29)	64(59.81)	146(84.39)	188(75.81)
Black or African American	46(13.45)	26(14.36)	142(28.92)	127(21.42)	169(24.21)	154(20.1)	186(22.12)	42(39.25)	27(15.61)	59(23.79)
Other/Unk/NR	1(0.29)	1(0.55)	17(3.46)	4(0.67)	5(0.72)	4(0.52)	5(0.59)	1(0.93)	0	1(0.4)
**Ethnicity (n, %)**										
Hispanic/Latino	1(0.29)	0	5(1.02)	3(0.51)	3(0.43)	4(0.52)	4(0.48)	0	1(0.58)	1(0.4)
Not Hispanic/Latino	337(98.54)	180(99.45)	483(98.37)	586(98.82)	691(99)	758(98.96)	833(99.05)	107(100)	172(99.42)	247(99.6)
Other/Unk/NR	4(1.17)	1(0.55)	3(0.61)	4(0.67)	4(0.57)	4(0.52)	4(0.48)	0	0	0
**Insurance (n, %)**										
Medicare	236(69.01)	126(69.61)	347(70.67)	405(68.3)	482(69.05)	554(72.32)	601(71.46)	77(71.96)	149(86.13)	196(79.03)
Medicaid	13(3.8)	8(4.42)	13(2.65)	19(3.2)	21(3.01)	21(2.74)	23(2.73)	3(2.8)	2(1.16)	4(1.61)
Commercial	92(26.9)	47(25.97)	104(21.18)	144(24.28)	170(24.36)	164(21.41)	190(22.59)	27(25.23)	20(11.56)	46(18.55)
Self-Insured/Unk/NR	1(0.29)	0	27(5.5)	25(4.22)	25(3.58)	27(3.52)	27(3.21)	0	2(1.16)	2(0.81)
**RUCA Code (n, %)**										
Missing	0	0	0	0	0	0	0	0	0	0
Metro Area	230(67.25)	123(67.96)	390(79.43)	432(72.85)	512(73.35)	564(73.63)	623(74.08)	82(76.64)	132(76.3)	191(77.02)
Rural Area	112(32.75)	58(32.04)	101(20.57)	161(27.15)	186(26.65)	202(26.37)	218(25.92)	25(23.36)	41(23.7)	57(22.98)
**ADI Group (n, %)**										
Missing	0	0	0	0	0	0	0	0	0	0
ADI Q1 (0-20%)	3(0.88)	3(1.66)	6(1.22)	7(1.18)	12(1.72)	9(1.17)	13(1.55)	5(4.67)	2(1.16)	6(2.42)

© 2025 Smeltzer MP et al. *JAMA Network Open*.

ADI Q2 (21-40%)	13(3.8)	8(4.42)	56(11.41)	33(5.56)	36(5.16)	57(7.44)	60(7.13)	4(3.74)	24(13.87)	27(10.89)
ADI Q3 (41-60%)	79(23.1)	40(22.1)	120(24.44)	127(21.42)	150(21.49)	167(21.8)	186(22.12)	24(22.43)	40(23.12)	59(23.79)
ADI Q4 (61-80%)	104(30.41)	55(30.39)	149(30.35)	217(36.59)	251(35.96)	266(34.73)	289(34.36)	34(31.78)	49(28.32)	72(29.03)
ADI Q5 (81-100%)	143(41.81)	75(41.44)	160(32.59)	209(35.24)	249(35.67)	267(34.86)	293(34.84)	40(37.38)	58(33.53)	84(33.87)
**Risk**										
**Smoking Status (n, %)**										
Active	255(74.56)	132(72.93)	152(30.96)	436(73.52)	502(71.92)	455(59.4)	503(59.81)	67(62.62)	19(10.98)	67(27.02)
Former	87(25.44)	49(27.07)	206(41.96)	157(26.48)	196(28.08)	311(40.6)	338(40.19)	40(37.38)	154(89.02)	181(72.98)
Never	0	0	126(25.66)	0	0	0	0	0	0	0
Unk	0	0	7(1.43)	0	0	0	0	0	0	0
**Pack Year (n, %*)**										
[0, 10)	0	0	68(18.99)	0	9(1.29)	0	10(1.19)	9(8.41)	0	10(4.03)
[10, 20)	0	0	42(11.73)	0	45(6.45)	0	52(6.18)	45(42.06)	0	52(20.97)
[20, 30)	13(3.8)	8(4.42)	16(4.47)	69(11.64)	79(11.32)	109(14.23)	109(12.96)	10(9.35)	40(23.12)	40(16.13)
[30, 40)	30(8.77)	18(9.94)	18(5.03)	64(10.79)	65(9.31)	93(12.14)	93(11.06)	3(2.8)	29(16.76)	29(11.69)
>= 40	299(87.43)	155(85.64)	68(18.99)	460(77.57)	493(70.63)	564(73.63)	564(67.06)	33(30.84)	104(60.12)	104(41.94)
Missing	0	0	146(40.78)	0	7(1)	0	13(1.55)	7(6.54)	0	13(5.24)
**Quit Year(n, %*)**										
[0, 15] years	87(100)	49(100)	40(19.42)	157(100)	196(100)	184(59.16)	197(58.28)	40(100)	27(17.53)	40(22.1)
[16, 25) years	0	0	32(15.53)	0	0	61(19.61)	64(18.93)	0	61(39.61)	64(35.36)
>= 25 years	0	0	91(44.17)	0	0	56(18.01)	64(18.93)	0	56(36.36)	64(35.36)
Missing	0	0	43(20.87)	0	0	10(3.22)	13(3.85)	0	10(6.49)	13(7.18)
**COPD (n, %)**										
No	146(42.69)	83(45.86)	366(74.54)	267(45.03)	316(45.27)	360(47)	407(48.39)	49(45.79)	93(53.76)	140(56.45)
Yes	196(57.31)	98(54.14)	125(25.46)	326(54.97)	382(54.73)	406(53)	434(51.61)	58(54.21)	80(46.24)	108(43.55)
**Comorbidity Distribution**										
Mean (SD)	1.64(1.27)	1.52(1.22)	1.37(1.33)	1.76(1.36)	1.78(1.38)	1.82(1.36)	1.79(1.36)	1.93(1.47)	2.03(1.37)	1.86(1.36)
Median (Q1-Q3)	1(1-2)	1(1-2)	1(0-2)	2(1-3)	2(1-3)	2(1-3)	2(1-3)	2(1-3)	2(1-3)	2(1-3)
(Min-Max)	(0 - 6)	(0 - 6)	(0 - 7)	(0 - 7)	(0 - 7)	(0 - 7)	(0 - 7)	(0 - 6)	(0 - 6)	(0 - 6)
**Charlson comorbidity (n, %)**										
0	56(16.37)	33(18.23)	149(30.35)	103(17.37)	118(16.91)	120(15.67)	137(16.29)	15(14.02)	17(9.83)	34(13.71)
1	176(51.46)	100(55.25)	200(40.73)	280(47.22)	319(45.7)	345(45.04)	381(45.3)	40(37.38)	65(37.57)	101(40.73)
2	110(32.16)	48(26.52)	142(28.92)	210(35.41)	261(37.39)	301(39.3)	323(38.41)	52(48.6)	91(52.6)	113(45.56)
**Prior history of Cancer (n, %)**										

© 2025 Smeltzer MP et al. *JAMA Network Open*.

No	264(77.19)	148(81.77)	354(72.1)	452(76.22)	501(71.78)	536(69.97)	596(70.87)	51(47.66)	84(48.55)	144(58.06)
Yes	78(22.81)	33(18.23)	137(27.9)	141(23.78)	197(28.22)	230(30.03)	245(29.13)	56(52.34)	89(51.45)	104(41.94)
**Prior LUNG cancer (n, %)**										
No	78(22.81)	33(18.23)	110(22.4)	141(23.78)	150(21.49)	176(22.98)	189(22.47)	9(8.41)	35(20.23)	48(19.35)
Yes	0	0	27(5.5)	0	47(6.73)	54(7.05)	56(6.66)	47(43.93)	54(31.21)	56(22.58)
**Family history of cancer (n, %)**										
No	88(25.73)	49(27.07)	155(31.57)	142(23.95)	164(23.5)	191(24.93)	207(24.61)	22(20.56)	49(28.32)	65(26.21)
Yes	213(62.28)	105(58.01)	224(45.62)	389(65.6)	452(64.76)	496(64.75)	538(63.97)	65(60.75)	107(61.85)	149(60.08)
Status Unknown	41(11.99)	27(14.92)	112(22.81)	62(10.46)	82(11.75)	79(10.31)	96(11.41)	20(18.69)	17(9.83)	34(13.71)
**Family LUNG cancer (n, %)**										
No	135(39.47)	70(38.67)	171(34.83)	245(41.32)	284(40.69)	315(41.12)	344(40.9)	41(38.32)	70(40.46)	99(39.92)
Yes	78(22.81)	35(19.34)	53(10.79)	144(24.28)	168(24.07)	181(23.63)	194(23.07)	24(22.43)	37(21.39)	50(20.16)
**Cancer Histology (n, %)**										
Adenocarcinoma	139(40.64)	93(51.38)	259(52.75)	264(44.52)	320(45.85)	355(46.34)	396(47.09)	57(53.27)	91(52.6)	132(53.23)
Squamous cell ca	118(34.5)	54(29.83)	92(18.74)	168(28.33)	194(27.79)	217(28.33)	235(27.94)	27(25.23)	49(28.32)	67(27.02)
Adenosquamous ca	1(0.29)	1(0.55)	3(0.61)	4(0.67)	5(0.72)	6(0.78)	7(0.83)	1(0.93)	2(1.16)	3(1.21)
Large cell	9(2.63)	7(3.87)	11(2.24)	17(2.87)	20(2.87)	19(2.48)	21(2.5)	3(2.8)	2(1.16)	4(1.61)
Small cell Lung ca	51(14.91)	16(8.84)	52(10.59)	77(12.98)	89(12.75)	94(12.27)	102(12.13)	12(11.21)	17(9.83)	25(10.08)
Other	23(6.73)	10(5.52)	69(14.05)	62(10.46)	69(9.89)	73(9.53)	78(9.27)	7(6.54)	11(6.36)	16(6.45)
Unk/NR	1(0.29)	0	5(1.02)	1(0.17)	1(0.14)	2(0.26)	2(0.24)	0	1(0.58)	1(0.4)
**Primary Tumor Size (cm)**										
Missing	21(6.14)	7(3.87)	26(5.3)	24(4.05)	29(4.15)	34(4.44)	39(4.64)	5(4.67)	10(5.78)	15(6.05)
Mean (SD)	2.47(1.85)	2.43(1.73)	3.65(2.51)	3.44(2.69)	3.41(2.68)	3.33(2.57)	3.33(2.56)	3.27(2.62)	2.94(2.06)	3.06(2.21)
Median (Q1-Q3)	1.9(1.3-3)	1.95(1.3-2.98)	3(1.8-4.9)	2.5(1.6-4.5)	2.5(1.6-4.5)	2.4(1.6-4.4)	2.45(1.6-4.3)	2.2(1.52-4.18)	2.2(1.5-3.8)	2.2(1.6-3.9)
(Min, Max)	(0.3 - 13.5)	(0.3 - 13.5)	(0.5 - 16.1)	(0.4 - 30)	(0.4 - 30)	(0.4 - 30)	(0.4 - 30)	(0.6 - 14.5)	(0.6 - 10)	(0.6 - 14.5)
**Clinical Stage (n, %)**										
Stage I	197(57.6)	105(58.01)	211(42.97)	261(44.01)	308(44.13)	357(46.61)	388(46.14)	47(43.93)	96(55.49)	127(51.21)
Stage II	22(6.43)	13(7.18)	32(6.52)	45(7.59)	59(8.45)	66(8.62)	74(8.8)	14(13.08)	21(12.14)	29(11.69)
Stage III	51(14.91)	29(16.02)	91(18.53)	137(23.1)	157(22.49)	166(21.67)	179(21.28)	21(19.63)	29(16.76)	42(16.94)
Stage IV	61(17.84)	29(16.02)	153(31.16)	145(24.45)	168(24.07)	171(22.32)	192(22.83)	24(22.43)	26(15.03)	47(18.95)
Unk/NR	11(3.22)	5(2.76)	4(0.81)	5(0.84)	6(0.86)	6(0.78)	8(0.95)	1(0.93)	1(0.58)	3(1.21)
**Clinical T Category (n, %)**										
cTX, cT0, cTis	6(1.75)	1(0.55)	9(1.83)	7(1.18)	10(1.43)	14(1.83)	16(1.9)	3(2.8)	7(4.05)	9(3.63)

© 2025 Smeltzer MP et al. *JAMA Network Open*.

cT1	228(66.67)	121(66.85)	224(45.62)	312(52.61)	368(52.72)	415(54.18)	450(53.51)	57(53.27)	103(59.54)	138(55.65)
cT2	43(12.57)	27(14.92)	101(20.57)	95(16.02)	105(15.04)	119(15.54)	129(15.34)	10(9.35)	24(13.87)	34(13.71)
cT3	25(7.31)	14(7.73)	52(10.59)	70(11.8)	84(12.03)	89(11.62)	98(11.65)	14(13.08)	19(10.98)	28(11.29)
cT4	30(8.77)	14(7.73)	100(20.37)	104(17.54)	126(18.05)	123(16.06)	141(16.77)	23(21.5)	19(10.98)	37(14.92)
Unk/NR	10(2.92)	4(2.21)	5(1.02)	5(0.84)	5(0.72)	6(0.78)	7(0.83)	0	1(0.58)	2(0.81)
**Clinical N Category (n, %)**										
cN0	229(66.96)	124(68.51)	319(64.97)	363(61.21)	437(62.61)	492(64.23)	545(64.8)	75(70.09)	129(74.57)	182(73.39)
cN1	20(5.85)	10(5.52)	16(3.26)	37(6.24)	41(5.87)	41(5.35)	44(5.23)	4(3.74)	4(2.31)	7(2.82)
cN2	45(13.16)	22(12.15)	89(18.13)	123(20.74)	140(20.06)	149(19.45)	158(18.79)	17(15.89)	26(15.03)	35(14.11)
cN3	38(11.11)	21(11.6)	63(12.83)	65(10.96)	75(10.74)	78(10.18)	87(10.34)	11(10.28)	13(7.51)	22(8.87)
Unk/NR	10(2.92)	4(2.21)	4(0.81)	5(0.84)	5(0.72)	6(0.78)	7(0.83)	0	1(0.58)	2(0.81)
**Clinical M Category (n, %)**										
cM0	270(78.95)	147(81.22)	323(65.78)	437(73.69)	518(74.21)	583(76.11)	635(75.51)	82(76.64)	146(84.39)	198(79.84)
cM1a	12(3.51)	7(3.87)	50(10.18)	32(5.4)	37(5.3)	39(5.09)	43(5.11)	5(4.67)	7(4.05)	11(4.44)
cM1b	16(4.68)	10(5.52)	37(7.54)	41(6.91)	48(6.88)	44(5.74)	52(6.18)	8(7.48)	3(1.73)	11(4.44)
cM1c	34(9.94)	13(7.18)	77(15.68)	78(13.15)	90(12.89)	94(12.27)	104(12.37)	12(11.21)	16(9.25)	26(10.48)
Unk/NR	10(2.92)	4(2.21)	4(0.81)	5(0.84)	5(0.72)	6(0.78)	7(0.83)	0	1(0.58)	2(0.81)

© 2025 Smeltzer MP et al. *JAMA Network Open*.

eFigure 1. Numbers Eligible and with Lung Cancer by Group

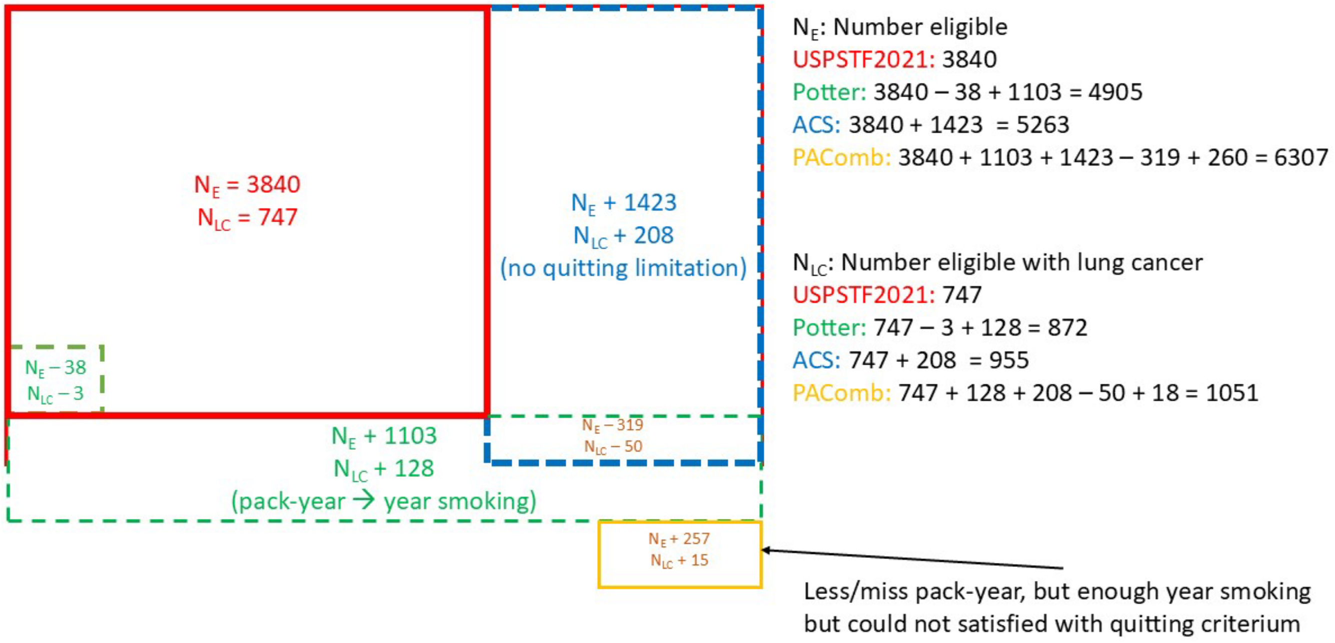

Number eligible and number with lung cancer by USPSTF2021, Potter, ACS Criteria, or Potter+ACS (PAComb) including overlap between groups.

© 2025 Smeltzer MP et al. *JAMA Network Open*.

eFigure 2. Area Deprivation Index (ADI) Quartiles in Enrollees and Lung Cancer By Eligibility Group

eFigure 2A. Area Deprivation Index (ADI) Quartiles in Enrollees by Eligibility Group

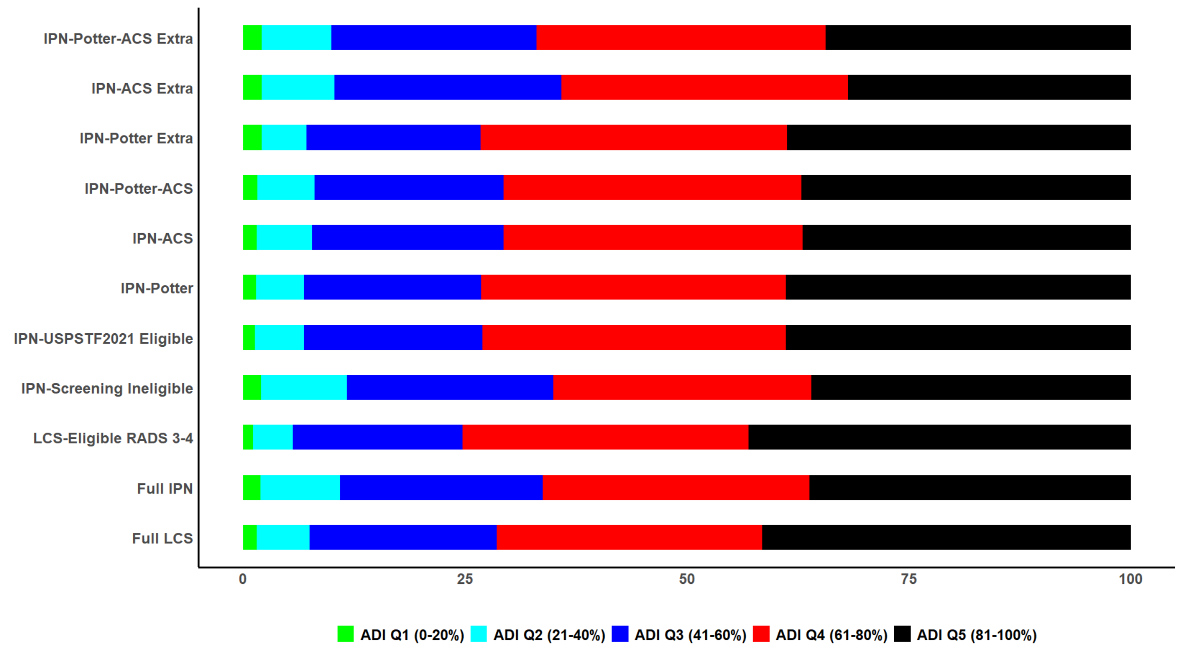

eFigure 2B. Area Deprivation Index (ADI) Quartiles in Lung Cancer Patients By Eligibility Group

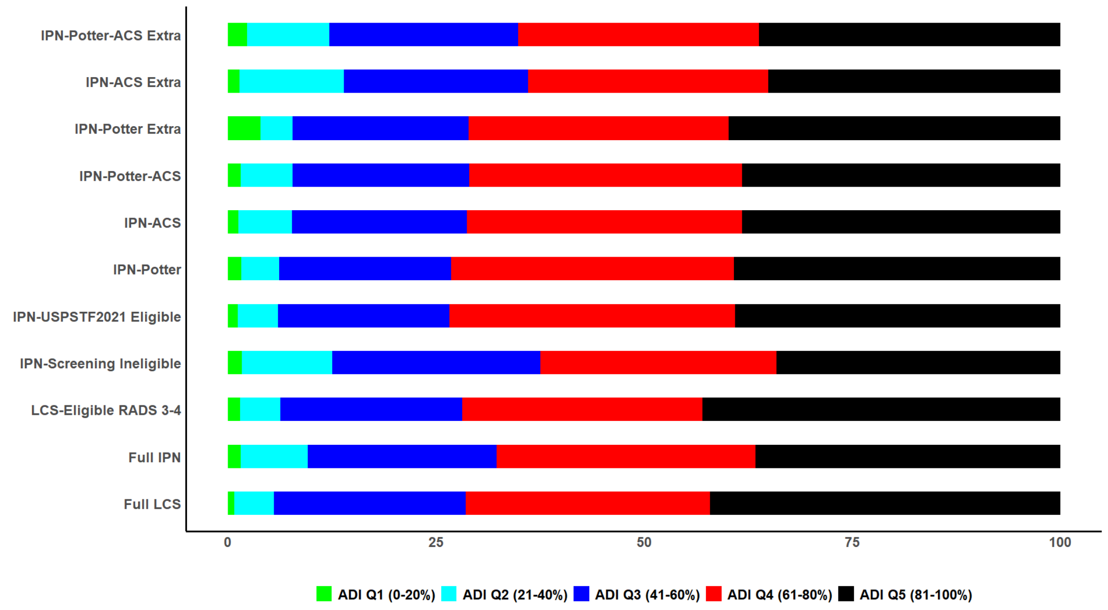

© 2025 Smeltzer MP et al. *JAMA Network Open*.

eFigure 3. Proportions of Females and Black Individuals Diagnosed With Lung Cancer by Pack-Years of Smoking Exposure and Years of Smoking Exposure

eFigure 3A. Proportions of Females Diagnosed with Lung Cancer by Pack Years of Smoking Exposure

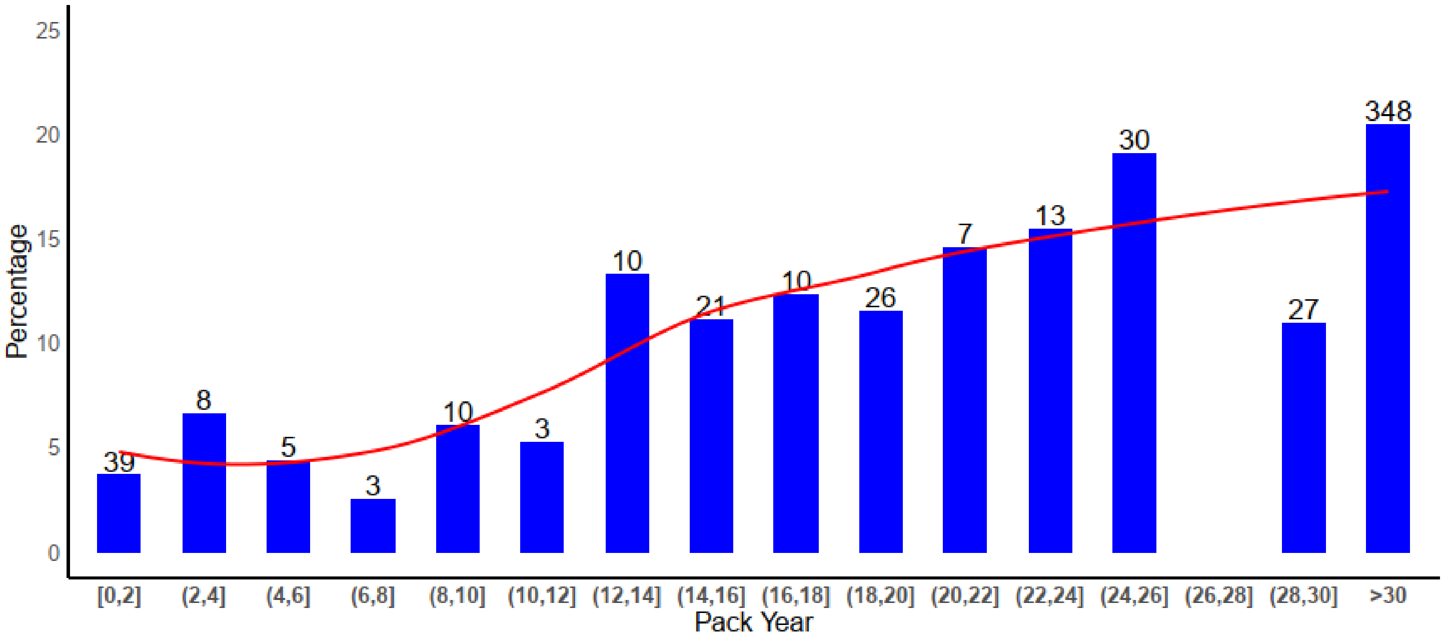

© 2025 Smeltzer MP et al. *JAMA Network Open*.

eFigure 3B. Proportions of Females Diagnosed with Lung Cancer by Years of Smoking Exposure

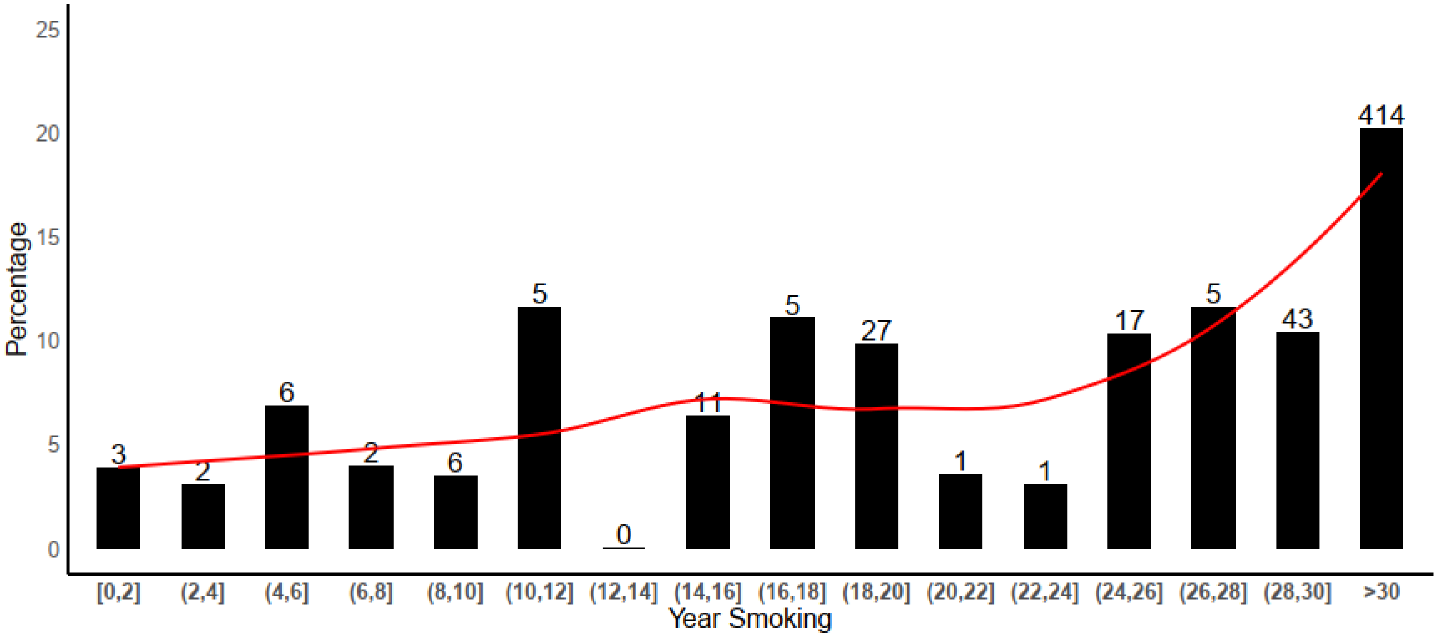

eFigure 3C. Proportions of Black Individuals Diagnosed with Lung Cancer by Pack Years of Smoking Exposure

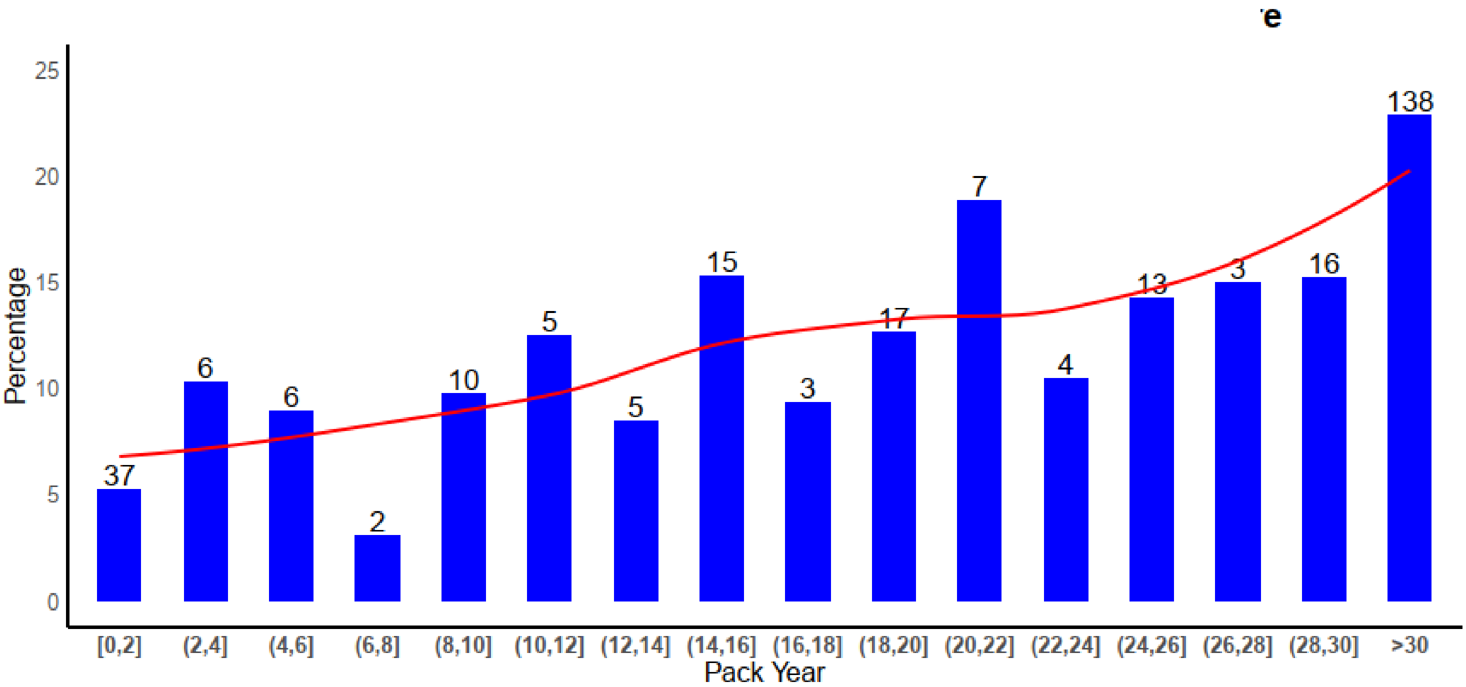

© 2025 Smeltzer MP et al. *JAMA Network Open*.

eFigure 3D. Proportions of Black Individuals Diagnosed with Lung Cancer by Years of Smoking Exposure

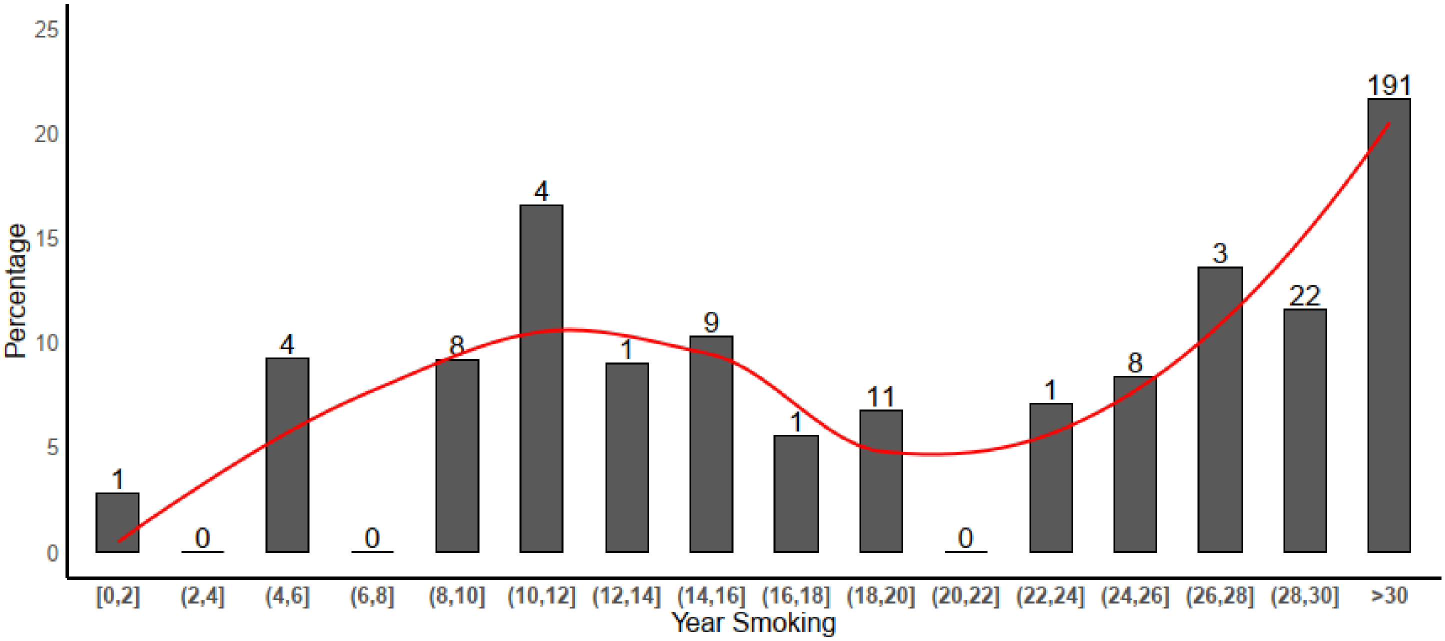

© 2025 Smeltzer MP et al. *JAMA Network Open*.
