# Supplementary material for: Reducing Smoking Requirements for Lung Screening to Address Health Disparities in a Community Cohort
Source: JAMA Netw Open. 2025 Jun 24;8(6):e2517149. doi: 10.1001/jamanetworkopen.2025.17149 (PMC12188365; doi:10.1001/jamanetworkopen.2025.17149)
Supplement: Supplement 2. — Data Sharing Statement [file jamanetwopen-e2517149-s002.pdf]

Data Sharing Statement

Smeltzer. Reducing Smoking Requirements for Lung Screening to Address Health Disparities in a Community Cohort. *JAMA Netw Open*. Published June 24, 2025. doi:10.1001/jamanetworkopen.2025.17149

Data
Data available: No

Additional Information
Explanation for why data not available: The data used in the study are derived from a limited dataset within the Baptist IRB approved REDCap database titled ‘Detecting Early Lung Cancer (DELUGE) in the Mississippi Delta cohort.’ Summary level data may be shared upon request if approved through the Baptist legal and regulatory channels.
